# Are disease reservoirs special? Taxonomic and life history characteristics

**DOI:** 10.1371/journal.pone.0180716

**Published:** 2017-07-13

**Authors:** Benjamin T. Plourde, Tristan L. Burgess, Evan A. Eskew, Tara M. Roth, Nicole Stephenson, Janet E. Foley

**Affiliations:** 1 Department of Medicine and Epidemiology, School of Veterinary Medicine, University of California, Davis, California, United States of America; 2 Karen C. Drayer Wildlife Health Center, School of Veterinary Medicine, University of California, Davis, California, United States of America; 3 Graduate Group in Ecology, University of California, Davis, California, United States of America; 4 EcoHealth Alliance, New York, New York, United States of America; CSIRO, AUSTRALIA

## Abstract

Pathogens that spill over between species cause a significant human and animal health burden. Here, we describe characteristics of animal reservoirs that are required for pathogen spillover. We assembled and analyzed a database of 330 disease systems in which a pathogen spills over from a reservoir of one or more species. Three-quarters of reservoirs included wildlife, and 84% included mammals. Further, 65% of pathogens depended on a community of reservoir hosts, rather than a single species, for persistence. Among mammals, the most frequently identified reservoir hosts were rodents, artiodactyls, and carnivores. The distribution among orders of mammalian species identified as reservoirs did not differ from that expected by chance. Among disease systems with high priority pathogens and epidemic potential, we found birds, primates, and bats to be overrepresented. We also analyzed the life history traits of mammalian reservoir hosts and compared them to mammals as a whole. Reservoir species had faster life history characteristics than mammals overall, exhibiting traits associated with greater reproductive output rather than long-term survival. Thus, we find that in many respects, reservoirs of spillover pathogens are indeed special. The described patterns provide a useful resource for studying and managing emerging infectious diseases.

## Introduction

Since the earliest documented epidemics of plague, leptospirosis, viral hemorrhagic fevers, and rabies, we have known that humans and our domestic animals can become ill after contact with other animals [1]. Most animal pathogens can infect multiple host species, and pathogen spillover from one host species to another is common [2]. Pathogen spillover has been defined as scenarios in which disease occurrence in a focal population depends on a distinct reservoir source that maintains the pathogen indefinitely [3]. Thus, controlling spillover diseases is complicated by the need to manage not only cases in the target population but also transmission interfaces and reservoir populations. Broadly, a disease reservoir is the source of new cases in a target population. A better understanding of the types of species that form reservoirs will therefore facilitate the management of many emerging infectious diseases.

However, what precisely constitutes a reservoir has been the subject of debate [4–6]. Haydon et al. [6] defined a reservoir as “one or more epidemiologically connected populations or environments in which the pathogen can be permanently maintained and from which infection is transmitted to the defined target population.” Following this definition, one pathogen may cause disease in multiple target populations, and the reservoir for each target population can be different. To identify reservoirs, researchers must find evidence of natural infection and spillover [7]. Here, we follow the Haydon et al. [6] definition to study reservoirs of spillover pathogens.

A number of recent studies have attempted to answer the question, “Are reservoirs special?”, with the goal of identifying likely disease sources [8,9]. Ungulates, carnivores, and rodents are the source of most zoonoses [2], yet recent outbreaks suggest that bats may be a uniquely important reservoir of human pathogens [8,10]. Reservoir species with fast life history characteristics appear to have higher reservoir competence [11] and contribute disproportionately to cases of some zoonotic diseases [12,13]. In a study of reservoirs for tick-borne zoonotic diseases, the mammalian species with the fastest life history traits also exhibited the highest reservoir competence [13]. Species with high reproductive output produce a large number of naïve host individuals that can sustain pathogens, even those that induce long-lasting host immunity. For example, the persistence of classical swine fever in pigs and wild boar has been attributed, in part, to high birth rates that maintain a large population of susceptible individuals [14]. Further, because of trade-offs between pace of life and the immune system investment, fast-lived species may exhibit greater reservoir competence through a proneness to acquire, maintain, and transmit pathogens [15,16].

To summarize characteristics of reservoirs and determine what makes reservoirs special, we assembled and analyzed a database of pathogens, their targets, and their known reservoirs to address the following questions: 1) What are the most represented taxa among reservoirs of spillover pathogens? 2) What are the characteristics and common taxa of reservoirs for pathogens that are zoonotic “high priority” (defined below) or have epidemic potential? And 3) Are mammalian reservoir species distinct in their life history traits compared to all mammals? Given the strong research bias in favor of human and domestic animal pathogens, we expect that most known disease reservoirs will include mammals. We also hypothesize that, more often than not, reservoirs will include multiple species and wildlife. Because past studies suggested that faster-lived species may be more competent hosts, we predict that reservoir species will exhibit faster life histories.

## Methods

### Database construction

We conducted a comprehensive literature search for pathogens that infect multiple host species and for which a reservoir is known or suggested, expanding on previously published lists [17–19] (S1 Dataset). Searches were conducted using Google Scholar and PubMed with search terms including the scientific names and synonyms of pathogens along with one or more of the following terms: reservoir, reservoir host, epidemiology, ecology, source, spillover, cases, host range, outbreak, and epidemic. We included any pathogen that spills over from one or more non-human animal species to another and causes clinical disease in the recipient (target) species. If, in addition to requiring an animal reservoir host, the pathogen also requires time developing in an external environment, then the environment was included as part of the reservoir. Each record in the resulting database is a unique combination of pathogen, target, reservoir, and geographic region, and is henceforth termed a “disease system.” Disease systems were excluded if there was weak or disputed evidence for the ability of the pathogen to cause disease in the target. For example, Sangassou virus, a recently discovered hantavirus in West Africa, has been found in its rodent host and neutralizing antibodies have been found in human fever patients; however, it was excluded from our analysis because the evidence remains inconclusive that it is the cause of human disease [20]. We also excluded any disease system for which the loss of the reservoir would not be likely to eliminate disease in the target because, following established definitions of reservoir and spillover, this could not truly be a reservoir. This most often occurred when spillover was well documented, but populations of the target species alone were sufficient for pathogen persistence.

A species was implicated as part of a pathogen reservoir if there were data showing infection of that species in nature (e.g., molecular detection, culture, and/or visual observation) and an epidemiological connection to the target host population (e.g., identification of risk factors or blood meal analysis). We assigned a confidence level to the evidence in support of each reservoir in each disease system: low confidence indicated that there was only evidence of natural infection but no epidemiological support for spillover, medium confidence required evidence of infection and epidemiological support for spillover, and high confidence corresponded to near certainty in reservoir identity (S2 Text). Disease systems with low confidence in reservoir identity were not included in further analyses.

We classified disease systems by pathogen taxon (bacteria, viruses, protozoa, fungi, oomycetes, or arthropods), geographic region (continent), and transmission type, which included one or more of: direct contact, vector-borne, trophic (including food- and water-borne transmission), or other indirect contact (such as contaminated surfaces). Systems with prion pathogens were excluded from this study.

We classified reservoirs as single species, multiple species, or complex. Disease systems with single species reservoirs require only a single species, excluding arthropod vectors, for maintenance of the pathogen. For example, most species of hantavirus were found to only depend on a single rodent species for persistence. If genetic evidence suggests distinct pathogen populations amongst different reservoir host populations, the pathogen may be maintained by multiple, single species reservoirs. In contrast, multiple species reservoirs comprise a community of more than one host species where the chronological order in which hosts acquire infection does not affect reservoir capacity and pathogen populations are not distinct between host species within the community. *Trypanosoma cruzi* uses a multiple species reservoir of sympatric opossums, armadillos, and coatis that together maintain a single pathogen population. A pathogen maintained by a complex reservoir completes each generation across multiple species and/or environments in a chronological order. Complex reservoirs are common to many helminth and protozoan parasites. This is illustrated by the life cycle of *Toxoplasma gondii*, which requires a suitable external environment, followed by a rodent host, followed by a feline host.

For vector-borne pathogens, we followed convention by defining the reservoir as solely the vertebrate hosts involved in pathogen maintenance. Arthropod vectors were only included in the reservoir cell if they alone comprised the reservoir. For example, Chandipura virus is maintained in populations of sandflies without the involvement of vertebrate blood meal hosts [21]. For all systems with vector-borne transmission we recorded the type of vector (e.g., tick, mosquito) and whether the vector is also required for pathogen persistence as separate data fields.

To classify pathogens depending on single versus multiple-species reservoirs, we created the “community-dependent” classification. Every disease system that included more than one species between the reservoir and required vectors was classified as community-dependent.

Targets and reservoirs were classified as one or more of humans (targets only), wildlife, domestic animals, livestock, companion animals, and the environment (reservoirs only). Reservoirs were separately classified by the major animal taxa they included as one or more of amphibians, annelids, arthropods, birds, fish, helminths, mammals, mollusks, and reptiles. Further, we recorded the mammalian orders, as given by Wilson and Reeder [22], associated with each reservoir when relevant.

### Mammalian reservoir representation by order

We created a list of the unique mammal species identified in the reservoirs of disease systems. We asked whether the unique reservoir host species we identified tended to occur in particular, non-random mammalian orders by comparing the frequency with which each order is represented in our data against the true distribution of species among mammalian orders. The expected representation of each order was calculated using the total number of species from *Mammal Species of the World* [22]. We calculated residuals for each comparison and transformed them into Pearson’s standardized residuals for comparisons among orders of different sizes. A chi-squared test was used to determine if the observed distribution was different from the true distribution of species among mammalian orders.

### Epidemic potential zone

We classified each disease system into one of three epidemic potential zones, based on the pathogen’s estimated basic reproductive number (R_0_) in the target. The zones are as follows: dead-end (D) for an R_0_ equal to zero, stuttering chains (S) for an R_0_ greater than zero but less than one, and epidemic potential (E) for an R_0_ greater than one. We inferred the most likely zone for each disease system based on evidence of intraspecific transmission in the target species. We gathered evidence through a literature search focused on epidemiology, transmission, and reports of cases and outbreaks. We aimed to assign the system to the zone that best describes the normal pattern of transmission following spillover. For example, if most spillover events result in a single case, but abnormal secondary transmission events are reported, the system was classified as dead-end. This was the case for many dead-end pathogens transmitted to humans, but for which occasional nosocomial or transplant-associated transmissions are known.

### High priority zoonotic pathogens

We used an H-index method for creating subsets of pathogens and their disease systems of particular significance for human health, which we call high priority zoonotic pathogens (HPZPs) [23]. This method estimates the societal attention paid to zoonotic pathogens by quantifying references to them in scientific literature. The H-index is the maximum number (*h*) for which *h* publications meeting some criteria (here, referring to a specific pathogen) have ≥ *h* citations [24].

First, we looked up H-indices using Web of Science [25] as of September 2015 for a comprehensive list of human pathogens from Taylor et al. [18]. Secondly, because there are now human pathogens in our database that were not listed by Taylor et al. [18], we followed the methods of McIntyre et al. [26] to calculate the H-index by creating search terms including the pathogen name in quotation marks, any synonyms listed by the NCBI Taxonomy Database, and virus acronyms, if applicable [23,26,27]. To create the HPZP subsets, we found the 75th and 90th percentile H-indices of the Taylor et al. list and used these thresholds to define the Top 25% (T25) and Top 10% (T10) priority subsets of our disease systems. All systems with an H-index above the 75th percentile were included in the T25 subset as were all above the 90th percentile in the T10 subset.

### Disease system statistics

We analyzed six disease system subsets in addition to the full dataset: human-target systems, top 25% HPZPs, top 10% HPZPs, dead-end transmission, stuttering chains transmission, and epidemic potential transmission. Disease systems within each subset were summarized by pathogen type, target, transmission type, and region. We summarized the reservoirs by type, category, taxon, and mammalian order. Fisher's exact tests were used to assess the association between categorical variables.

### Mammalian reservoir life history traits

We used the list of unique mammalian reservoir species in our database to address the question of whether mammalian reservoir species have faster or slower life history traits than mammals as a whole. A database of mammalian life history traits was assembled using PanTHERIA [28], mapped to the third edition of *Mammal Species of the World* [22], as the starting point. We included the following traits: adult body mass (g), age at sexual maturity (days), gestation length (days), interbirth interval (days), litter size, neonate body mass (g), maximum longevity, number of litters per year, weaning age (days), and weaning mass (g). Recognizing that PanTHERIA is incomplete and biased towards well-studied taxa, we augmented our database using other compilations of mammalian life history data [29–33]. Male and female ages at sexual maturity are reported separately in the AnAge database [33] but were averaged for inclusion in our dataset. For any species with missing data for all ten life history traits, we searched the literature for adult body mass, which is correlated with the other traits [34]. Where there was no report of a species’ adult body mass, we used the adult body mass of the most closely-related species for which data were available.

We imputed estimates for missing values in the mammalian reservoir life history trait database by random forest imputation using the R package ‘missForest’ [35,36]. We constructed a mammalian phylogeny including order, family, genus, and species using the R package ‘ape’ [37]. This phylogeny was decomposed into phylogenetic eigenvectors [38]. Following Penone et al. [39], the first 10 eigenvectors were used as predictors and all life history variables were log-transformed. In short, missForest generated a random forest based on the known life history and phylogenetic values in our database to calculate estimates for each missing value [35,36].

After completing the trait database via imputation, we narrowed our focus to seven life history traits that comprise the fast-slow life history continuum: adult body mass, age at sexual maturity, gestation length, interbirth interval, litter size, neonate body mass, and weaning age [34]. Following Bielby et al. [34], we log-transformed all trait values and computed mass-corrected trait residuals by regressing every other trait on adult body mass in order to investigate the role of body mass-independent variation in life history traits. Analysis of the six remaining traits was conducted using these mass-corrected residuals.

We used permutation tests to compare residual trait values between all mammals and our identified mammalian reservoir species (178 unique species). Briefly, we used the R statistical software to randomly generate a sample of 178 mammalian species from our complete mammalian life history trait database (5,416 species) having the same representation by order as our identified mammalian reservoir species list [40]. We then calculated the mean body mass-corrected residual value for a given trait using that randomly generated sample of mammalian species. This process was repeated 1,000 times to generate a distribution of mean residual trait values. The resulting distribution of 1,000 mean values was then compared to our observed mean residual trait value for mammalian reservoir species, allowing us to identify life history traits for which mammalian reservoir species have significantly different values than mammals as a whole. This analysis was repeated with all six life history traits, considering only mammalian reservoirs from systems with humans listed as a target (155 unique species).

Using the same six mass-corrected trait residuals, we conducted a principal components analysis using the ‘prcomp’ function in R [40]. The resulting principal component (PC) scores represented new composite variables that might distinguish mammalian reservoir species from all mammals along the fast-slow continuum. We therefore conducted permutation tests as described above with PC scores as the variables of interest instead of trait residuals.

## Results

### Data collection and summary

The initial search for reservoirs of spillover pathogens returned 752 disease systems, of which 422 disease systems did not meet one or more inclusion criteria (S2 Dataset). This left 330 disease systems for which the available evidence strongly supports the implicated reservoir (S1 Dataset). The pathogen species or strains involved in these 330 disease systems include: 112 viruses, 88 bacteria, 64 helminths, 45 protozoans, 10 arthropods, 10 fungi, and one oomycete (Table 1). Pathogens of disease systems for which humans are the target (n = 261) were similarly distributed (Table 1). Among the 330 disease systems, 299 unique pathogens were represented, with 237 of these being zoonotic. The number of total disease systems and unique pathogens were not the same because, for some pathogens, there were different reservoirs in different regions, leading to more than one disease system for those pathogens.

**Table 1:** Summary of all disease systems and subsets by pathogen type.

		Epidemic Potential Zone*				High Priority Zoonotic Pathogens†	
Pathogen Type	All Systems (n = 330)	Dead-End (220)	Stuttering Chains (44)	Epidemic Potential (66)	Human Target (261)	Top 25% (109)	Top 10% (45)
Arthropods	3.03% (10)	2.73% (6)	0.00% (0)	6.06% (4)	2.68% (7)	0.00% (0)	0.00% (0)
Bacteria	26.67% (88)	26.82% (59)	38.64% (17)	18.18% (12)	28.35% (74)	39.45% (43)	33.33% (15)
Fungi	3.03% (10)	3.18% (7)	0.00% (0)	4.55% (3)	2.68% (7)	0.92% (1)	2.22% (1)
Helminths	19.39% (64)	26.36% (58)	4.55% (2)	6.06% (4)	23.37% (61)	11.93% (13)	2.22% (1)
Oomycetes	0.30% (1)	0.00% (0)	0.00% (0)	1.52% (1)	0.00% (0)	0.00% (0)	0.00% (0)
Protozoans	13.64% (45)	10.00% (22)	27.27% (12)	16.67% (11)	11.49% (30)	15.60% (17)	24.44% (11)
Viruses	33.94% (112)	30.91% (68)	29.55% (13)	46.97% (31)	31.42% (82)	32.11% (35)	37.78% (17)

Each percentage is the proportion of disease systems that include each type of pathogen. Each system included exactly one pathogen and thus one pathogen type. Prions were excluded from this study. The number of systems is given in parentheses.

*The epidemic potential zone subsets represent the transmission potential of the pathogen in the target host population(s) following spillover. The zones are defined as follows: dead-end for a basic reproductive number (R_0_) nearly equal to zero, stuttering chains for an R_0_ greater than zero but less than one, and epidemic potential for an R_0_ greater than one.

^†^High priority zoonotic pathogen subsets were determined by estimating the pathogen’s representation in the scientific literature using the H-index. Each subset was created to include pathogens that are among the 25% (Top 25%) and 10% (Top 10%) most significant known human pathogens.

Overall, 79% of the disease systems included humans as a target. Domestic animals were a target host for 23%, including livestock in 16% and companion animals in 8%. Wildlife were among targets in 12% of disease systems. Disease systems were distributed globally with at least one-third found on each continent save Antarctica (S1 Table). North America had the highest representation (54%) and Oceania had the lowest (33%).

When disease systems were summarized by reservoir type, nearly half (49%) had single species reservoirs. Multiple species reservoirs accounted for 32% and complex reservoirs for 19% of systems (Table 2). Almost two-thirds of all reservoirs (65.2%) and 70.5% of reservoirs of human-target disease systems were classified as community-dependent, such that the pathogen requires two or more host species to persist (Table 2).

**Table 2:** Summary of all disease systems and subsets by reservoir type.

		Epidemic Potential Zone*				High Priority Zoonotic Pathogens†	
Reservoir Type	All Systems (n = 330)	Dead-End (220)	Stuttering Chains (44)	Epidemic Potential (66)	Human Target (261)	Top 25% (109)	Top 10% (45)
Single Species	49.09% (162)	44.09% (97)	54.55 (24)	62.12% (41)	43.68% (114)	46.79% (51)	60.00% (27)
Multiple Species	31.12% (106)	30.45% (67)	38.64% (17)	33.33% (22)	33.72% (88)	41.28% (45)	37.78% (17)
Complex	18.79% (62)	25.45% (56)	6.83% (3)	4.55% (3)	22.61% (59)	11.93% (13)	2.22% (1)
Community‡	65.15% (215)	69.55% (153)	61.36% (27)	53.03% (35)	70.50% (184)	70.64% (77)	68.89% (31)

Each system was classified as exactly one of single species, multiple species, or complex reservoir type. Complex reservoirs include a defined sequence of environments and/or host species that maintain the pathogen. Community dependence is an additional yes or no classification that could include systems categorized as any of the first three (see ‡). The number of systems is given in parentheses.

*The epidemic potential zone subsets represent the transmission potential of the pathogen in the target host population(s) following spillover. The zones are defined as follows: dead-end for a basic reproductive number (R_0_) nearly equal to zero, stuttering chains for an R_0_ greater than zero but less than one, and epidemic potential for an R_0_ greater than one.

^†^High priority zoonotic pathogen subsets were determined by estimating the pathogen’s representation in the scientific literature using the H-index. Each subset was created to include pathogens that are among the 25% (Top 25%) and 10% (Top 10%) most significant known human pathogens.

^‡^Community dependence is calculated as follows: number of systems with multiple species reservoirs + (number of complex reservoir systems—number of complex reservoirs with only one animal species) + number of non-arthropod single species reservoirs with a required arthropod vector.

Three-quarters of reservoirs included wildlife (n = 247) and 42% (n = 137) included at least one domestic animal (Table 3). Of the domestic animals, livestock were a part of the reservoir for 24% and companion animals for 20%. The distribution of reservoir categories within human-target disease systems was similar (Table 3).

**Table 3:** Summary of all disease systems and subsets by reservoir category.

		Epidemic Potential Zone*				High Priority Zoonotic Pathogens†	
Reservoir Category	All Systems (n = 330)	Dead-End (220)	Stuttering Chains (44)	Epidemic Potential (66)	Human Target (261)	Top 25% (109)	Top 10% (45)
Wildlife	74.85% (247)	76.36% (168)	63.64% (28)	77.27% (51)	75.48% (197)	74.31% (81)	77.78% (35)
Domestic Animals	41.52% (137)	40.45% (89)	54.55% (24)	36.36% (24)	41.76% (109)	39.45% (43)	33.33% (15)
Livestock	24.24% (80)	20.00% (44)	43.18% (19)	25.76% (17)	23.75% (62)	25.69% (28)	28.89% (13)
Companion Animals	20.00% (66)	23.18% (51)	15.91% (7)	12.12% (8)	21.46% (56)	16.51% (18)	4.44% (2)
Environment	10.00% (33)	13.18% (29)	4.55% (2)	3.03% (2)	12.64% (33)	6.42% (7)	2.22% (1)

Each reservoir was categorized by one or more of wildlife, domestic animals, livestock, companion animals, and environment. All domestic animal reservoir species were categorized as livestock, companion animals, or both, depending on which types of populations host the pathogen. Decisions between wildlife and/or livestock were made with the same criterion. The number of systems is given in parentheses.

*The epidemic potential zone subsets represent the transmission potential of the pathogen in the target host population(s) following spillover. The zones are defined as follows: dead-end for a basic reproductive number (R_0_) nearly equal to zero, stuttering chains for an R_0_ greater than zero but less than one, and epidemic potential for an R_0_ greater than one.

^†^High priority zoonotic pathogen subsets were determined by estimating the pathogen’s representation in the scientific literature using the H-index. Each subset was created to include pathogens that are among the 25% (Top 25%) and 10% (Top 10%) most significant known human pathogens.

Disease system reservoirs predominantly included mammals (84%) (Table 4). The next most-represented reservoir taxa were arthropods (42%), the environment (10%, see Table 3), birds (9%), and mollusks (7%). Fish, amphibians, reptiles, helminths, and annelids each appeared as reservoirs in 5% or less of the disease systems (Table 4).

**Table 4:** Summary of disease system reservoirs by major animal taxa.

		Epidemic Potential Zone*				High Priority Zoonotic Pathogens†	
Reservoir Taxon	All Systems (n = 330)	Dead-End (220)	Stuttering Chains (44)	Epidemic Potential (66)	Human Target (261)	Top 25% (109)	Top 10% (45)
Amphibian	3.03% (10)	3.18% (7)	0.00% (0)	4.55% (3)	2.68% (7)	0.00% (0)	0.00% (0)
Annelid	0.30% (1)	0.45% (1)	0.00% (0)	0.00% (0)	0.38% (1)	0.00% (0)	0.00% (0)
Arthropod‡	42.42% (140)	43.64% (96)	40.91% (18)	39.39% (26)	45.21% (118)	44.04% (48)	44.44% (20)
Bird	8.79% (29)	9.09% (20)	6.82% (3)	9.09% (6)	9.96% (26)	12.84% (14)	17.78% (8)
Fish	5.15% (17)	7.27% (16)	0.00% (0)	1.52% (1)	6.13% (16)	0.92% (1)	0.00% (0)
Helminth	0.91% (3)	1.36% (3)	0.00% (0)	0.00% (0)	0.00% (0)	0.00% (0)	0.00% (0)
Mammal	84.24% (278)	84.09% (185)	93.18% (41)	78.79% (52)	86.59% (226)	86.24% (94)	88.89% (40)
Mollusk	7.27% (24)	7.73% (17)	4.55% (2)	7.58% (5)	7.28% (19)	2.75% (3)	0.00% (0)
Reptile	2.42% (8)	3.18% (7)	2.27% (1)	0.00% (0)	3.07% (8)	0.00% (0)	0.00% (0)

Each reservoir was categorized by which of the listed animal taxa or environments it included. The data above show the percentage of systems whose reservoirs include at least one species belonging to the group. The number of systems is given in parentheses.

*The epidemic potential zone subsets represent the transmission potential of the pathogen in the target host population(s) following spillover. The zones are defined as follows: dead-end for a basic reproductive number (R_0_) nearly equal to zero, stuttering chains for an R_0_ greater than zero but less than one, and epidemic potential for an R_0_ greater than one.

^†^High priority zoonotic pathogen subsets were determined by estimating the pathogen’s representation in the scientific literature using the H-index. Each subset was created to include pathogens that are among the 25% (Top 25%) and 10% (Top 10%) most significant known human pathogens.

^‡^Includes arthropods listed in the reservoir field as well as those listed as vectors only.

Common mammalian orders found within reservoirs were rodents, artiodactyls, and carnivores (Table 5), each in the reservoir for 29%, 25%, and 25% of total disease systems, respectively. Bats were only found in the reservoir of eight systems (2.4%) (Table 5), all of which included a viral pathogen and humans as a target host. As a result, the percentage of systems with bat reservoirs increases substantially when considering only viral pathogen systems (7.1%) and viral pathogen systems with human targets (9.8%) (S6 Table). Domestic and peridomestic animals dominated the most common mammalian reservoir hosts including dogs (n = 44 systems), cats (n = 37), cattle (n = 32), pigs (n = 24), sheep (n = 22), rats (*Rattus rattus*, n = 18; *Rattus norvegicus*, n = 13), and goats (n = 16). The top eight mammalian reservoir host species for human-target systems were the same (S5 Table).

**Table 5:** Summary of mammalian reservoirs by orders represented.

		Epidemic Potential Zone*				High Priority Zoonotic Pathogens†	
Order	All Systems (n = 330)	Dead-End (220)	Stuttering Chains (44)	Epidemic Potential (66)	Human Target (261)	Top 25% (109)	Top 10% (45)
Artiodactyla	25.45% (84)	20.45% (45)	47.73% (21)	27.27% (18)	21.07% (55)	23.85% (26)	26.67% (12)
Carnivora	25.15% (83)	28.64% (63)	20.45% (9)	16.67% (11)	25.29% (66)	22.94% (25)	15.56% (7)
Cetacea	0.30% (1)	0.45% (1)	0.00% (0)	0.00% (0)	0.38% (1)	0.00% (0)	0.00% (0)
Chiroptera	2.42% (8)	1.36% (3)	2.27% (1)	6.06% (4)	3.07% (8)	4.59% (5)	4.44% (2)
Cingulata	0.91% (3)	0.45% (1)	4.55% (2)	0.00% (0)	1.15% (3)	1.83% (2)	4.44% (2)
Didelphimorphia	0.61% (2)	0.00% (0)	4.55% (2)	0.00% (0)	0.77% (2)	1.83% (2)	2.22% (1)
Diprotodontia	0.61% (2)	0.45% (1)	0.00% (0)	1.52% (1)	0.38% (1)	0.92% (1)	0.00% (0)
Erinaceomorpha	0.30% (1)	0.45% (1)	0.00% (0)	0.00% (0)	0.38% (1)	0.00% (0)	0.00% (0)
Hyracoidea	0.30% (1)	0.45% (1)	0.00% (0)	0.00% (0)	0.38% (1)	0.00% (0)	0.00% (0)
Lagomorpha	3.03% (10)	2.73% (6)	0.00% (0)	6.06% (4)	2.30% (6)	0.00% (0)	0.00% (0)
Perissodactyla	2.73% (9)	3.18% (7)	0.00% (0)	3.03% (2)	3.07% (8)	0.92% (1)	0.00% (0)
Pilosa	0.61% (2)	0.45% (1)	2.27% (1)	0.00% (0)	0.77% (2)	0.00% (0)	0.00% (0)
Primates	3.03% (10)	1.36% (3)	4.55% (2)	7.58% (5)	3.83% (10)	6.42% (7)	8.89% (4)
Rodentia	29.09% (96)	33.64% (74)	25.00% (11)	16.67% (11)	36.02% (94)	35.78% (39)	35.56% (16)
Soricomorpha	1.21% (4)	1.36% (3)	2.27% (1)	0.00% (0)	1.53% (4)	1.83% (2)	0.00% (0)

Each reservoir was categorized by the orders of mammals represented by the species it included. Orders reflect the hierarchy detailed in *Mammal Species of the World* [22]. The data above show the percentage of systems whose reservoirs include at least one species belonging to the order. The number of systems is given in parentheses. Unlisted mammalian orders were not found among reservoirs.

*The epidemic potential zone subsets represent the transmission potential of the pathogen in the target host population(s) following spillover. The zones are defined as follows: dead-end for a basic reproductive number (R_0_) nearly equal to zero, stuttering chains for an R_0_ greater than zero but less than one, and epidemic potential for an R_0_ greater than one.

^†^High priority zoonotic pathogen subsets were determined by estimating the pathogen’s representation in the scientific literature using the H-index. Each subset was created to include pathogens that are among the 25% (Top 25%) and 10% (Top 10%) most significant known human pathogens.

Among all disease systems, 178 unique mammal species were found within the reservoirs. Based on this list, rodents were the most represented order (n = 72 species, 40%), followed by carnivores (n = 26, 15%), artiodactyls (n = 24, 14%), and bats (n = 19, 11%). The 155 unique mammalian reservoirs in human-target disease systems followed a similar pattern, with all 72 unique rodent species (46%) in the database serving as reservoirs in human-target systems. In addition, 23 carnivore (15%) and 19 bat species (12%) were reservoir hosts of human pathogens. Artiodactyls were fourth-most represented with eight unique reservoir species (5%).

**Fig 1:**
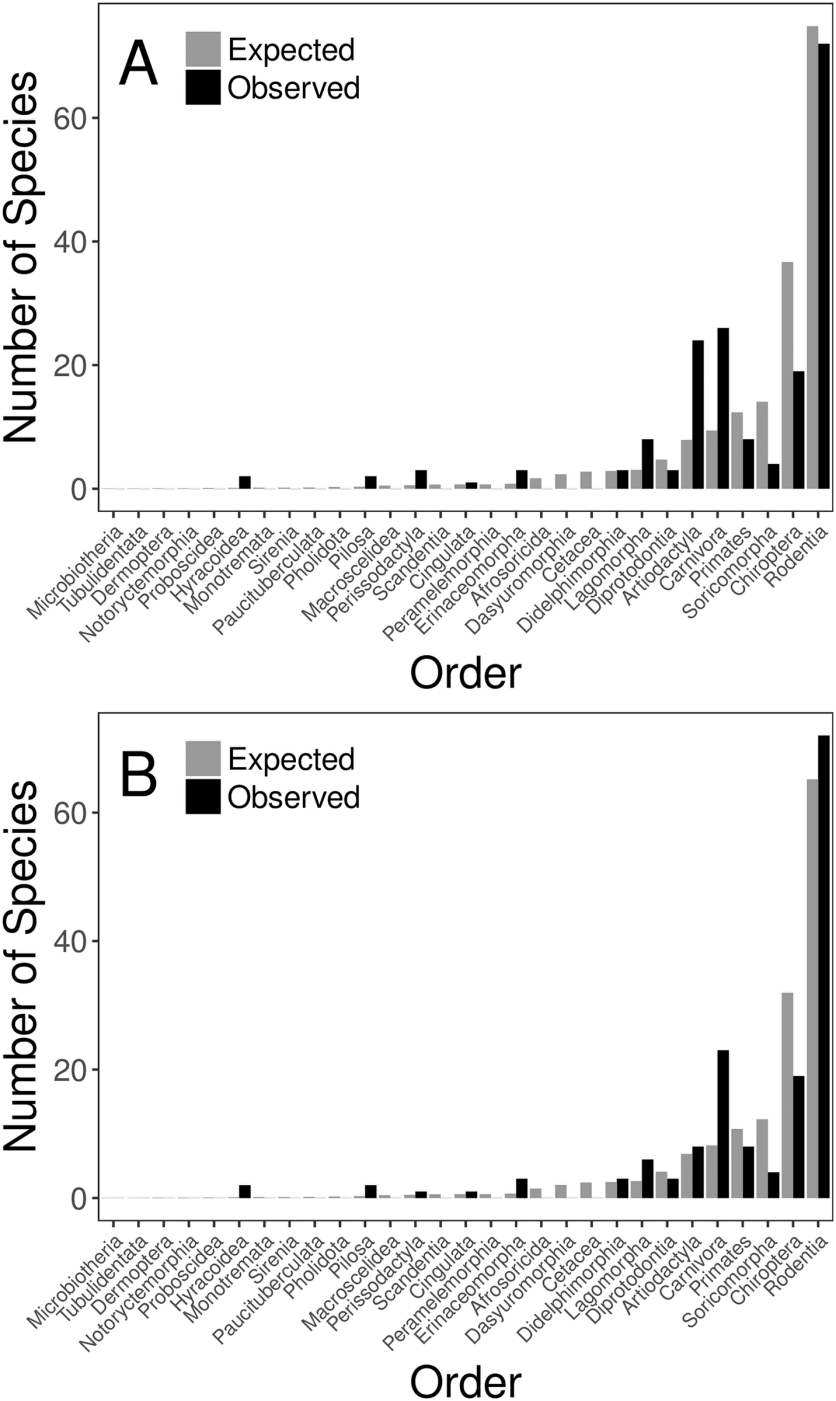
Expected versus observed representations of mammalian orders among reservoirs. The expected representations (gray bars) assume that orders are found among reservoir mammals in the same proportion as they are among all mammals (n = 5,416) listed by Wilson and Reeder [22]. Observed bars (black) show the number of unique mammalian species in each order that were identified as reservoir hosts in this study. (A) shows the results for all systems (n = 330) in which 178 mammalian species were found to be reservoir hosts. (B) shows results for the human target subset of systems (n = 261) in which 155 mammalian species were found to be reservoir hosts.

Among the six mammalian orders in which we identified at least five unique reservoir species, artiodactyls (standardized residual = +5.74), carnivores (+5.41), and lagomorphs (+2.86) were overrepresented compared to the proportion of mammalian species in each order (Fig 1A, S3 Table). Bats (standardized residual = -2.92), primates (-1.24), and rodents (-0.33) were underrepresented (Fig 1A, S3 Table). The same relationships were true for human-target pathogens, except that artiodactyls were only slightly overrepresented (+0.43) and rodents were overrepresented (+0.85) in these systems (Fig 1B, S4 Table). Among only viral pathogen systems, the number of bat reservoir species matched that expected by chance (19 expected, 19 observed) (S7 Table). However, bats were overrepresented for viral pathogen systems with human targets (16 expected, 19 observed) (S7 Table).

The observed distribution of mammalian orders among reservoirs did not significantly differ from expectation based on the number of species in each mammalian order (Pearson’s chi-squared test, χ^2^ = 245.53, p = 0.082), however, they were significantly different in the human-target system subset (χ^2^ = 252.78, p = 0.044).

### Epidemic potential zone

Two-thirds of disease systems were classified as dead-ends (D) (n = 220), 44 were classified as stuttering chains (S), and 66 had epidemic potential (E). The distribution of reservoir types among the three zones was significantly different (Fisher’s exact test, p < 0.001). Nearly all complex reservoirs were in disease systems where the pathogen dead-ends in the target hosts (56/62, 90.3%) (Table 2). The proportion of disease systems with single species reservoirs was highest in those with epidemic potential (D: 44.1%, S: 54.6%, E: 62.1%) (Table 2). Artiodactyls (p = 0.001) and livestock (p = 0.006) had increased representation in the S category (Tables 3 and 5). The proportion of disease systems with rodents in the reservoir decreased with epidemic potential (D: 33.6%, S: 25.0%, E: 16.7%) (Fisher’s exact test, p = 0.0214) (Table 5). The percentage of disease systems having bats (D: 1.4%, S: 2.3%, E: 6.1%) and primates (D: 1.4%, S: 4.6%, E: 7.6%) within the reservoir increased with epidemic potential (Table 5). However, the distribution of bat reservoirs amongst the zones was not significantly different (Fisher’s exact test, p = 0.083), while the distribution of primate reservoirs did significantly differ with epidemic potential (p = 0.019).

### High priority zoonotic pathogens

The 75^th^ percentile of H-indices for human pathogens on the Taylor et al. [18] list was 55, which was exceeded by 109 of the 261 human-target systems in our database (41.8%). These 109 systems made up the T25 subset. The 90^th^ percentile was 102.7, which was exceeded by 45 of the 261 systems (17.2%) to create the T10 subset. Compared to non-HPZP systems, the HPZP systems included proportionally more single species and multiple species reservoirs but fewer complex reservoirs (Fisher’s exact test, p = 0.001) (Table 2). The proportions of HPZP and non-HPZP systems found to be community-dependent were similar (non-HPZP: 70.4%, HPZP: 70.6%; Fisher’s exact test, p = 1) (Table 2).

Representation by mammals and arthropods in reservoirs was similar for HPZPs compared to all human-target systems (Table 4). Only mammals, arthropods, birds, and the environment were found in the T10 HPZP reservoirs. Three T25 HPZP reservoirs included mollusks and one included fish. Birds were more represented among HPZP systems than they were among all human-target systems (All: 10.0%, T25: 12.8%, T10: 17.8%). However, the proportion of HPZP systems with avian reservoirs did not significantly differ from that of non-HPZP systems (Fisher’s exact test, p = 0.212). Reptiles, amphibians, and annelids were not found in the reservoirs of either subset, and the environment was less represented in HPZP reservoirs when compared to non-HPZP reservoirs (Fisher’s exact test, p = 0.0133) (Table 4). Companion animals were also less represented among HPZP reservoirs, but the difference was not significant (Fisher’s exact test, p = 0.126) (Table 3).

There were also differences in the distribution of mammalian orders amongst the HPZP reservoirs. The representation of carnivores in the T25 subset was similar to all human-target systems (All: 25.3%, T25: 22.9%), but they were less represented in the T10 subset (15.6%) (Table 5). Primate representation increased in the HPZP subset reservoirs (All: 3.8%, T25: 6.4%, T10: 8.9%), however, there was not a significant difference between primate representation in HPZP versus non-HPZP reservoirs (Fisher’s exact test, p = 0.0993). Likewise, the contribution of bats increased slightly, but not significantly, among HPZPs (Fisher’s exact test, p = 0.284) (All: 3.1%, T25: 4.6%, T10: 4.4%). The following orders that were in the reservoir for at least one human-target pathogen did not appear in the reservoirs of the T10 HPZP subset: Cetacea, Diprotodontia, Erinaceomorpha, Hyracoidea, Lagomorpha, Perissodactyla, Pilosa, and Soricomorpha (Table 5).

### Mammalian reservoir life history traits

The PanTHERIA database was 25.8% complete for the ten life history traits we selected. After inclusion of data from other databases, this increased to 28.7%. The life history trait data were 76.6% complete for the mammalian species we identified as reservoirs. However, PanTHERIA was particularly data-deficient for domestic animal reservoirs, especially cats and cattle, which appeared as reservoir hosts in 37 and 32 of our disease systems, respectively. Cats, cattle, and the brown rat (*Rattus norvegicus*) had life history traits missing that were supplemented from the primary literature [41–43]. Six reservoir species (*Myodes rex*, *Apodemus argenteus*, *A*. *ponticus*, *Meriones sacramenti*, *Rhinolophus sinicus*, and *Presbytis melalophos*) were missing all life history traits. Adult body mass could be found in the primary literature for three of the species [44–46], and the others were extrapolated from closely-related species. The mass of *Apodemus flavicollis* was used for *A*. *ponticus*. The most closely related species of *Rhinolophus sinicus* and *Presbytis melalophos* could not be determined. For these two species, we used the average of reported body masses in each of their genera as an estimate. Once these supplemental data were included, life history trait data for reservoir species were 91.8% complete.

**Fig 2:**
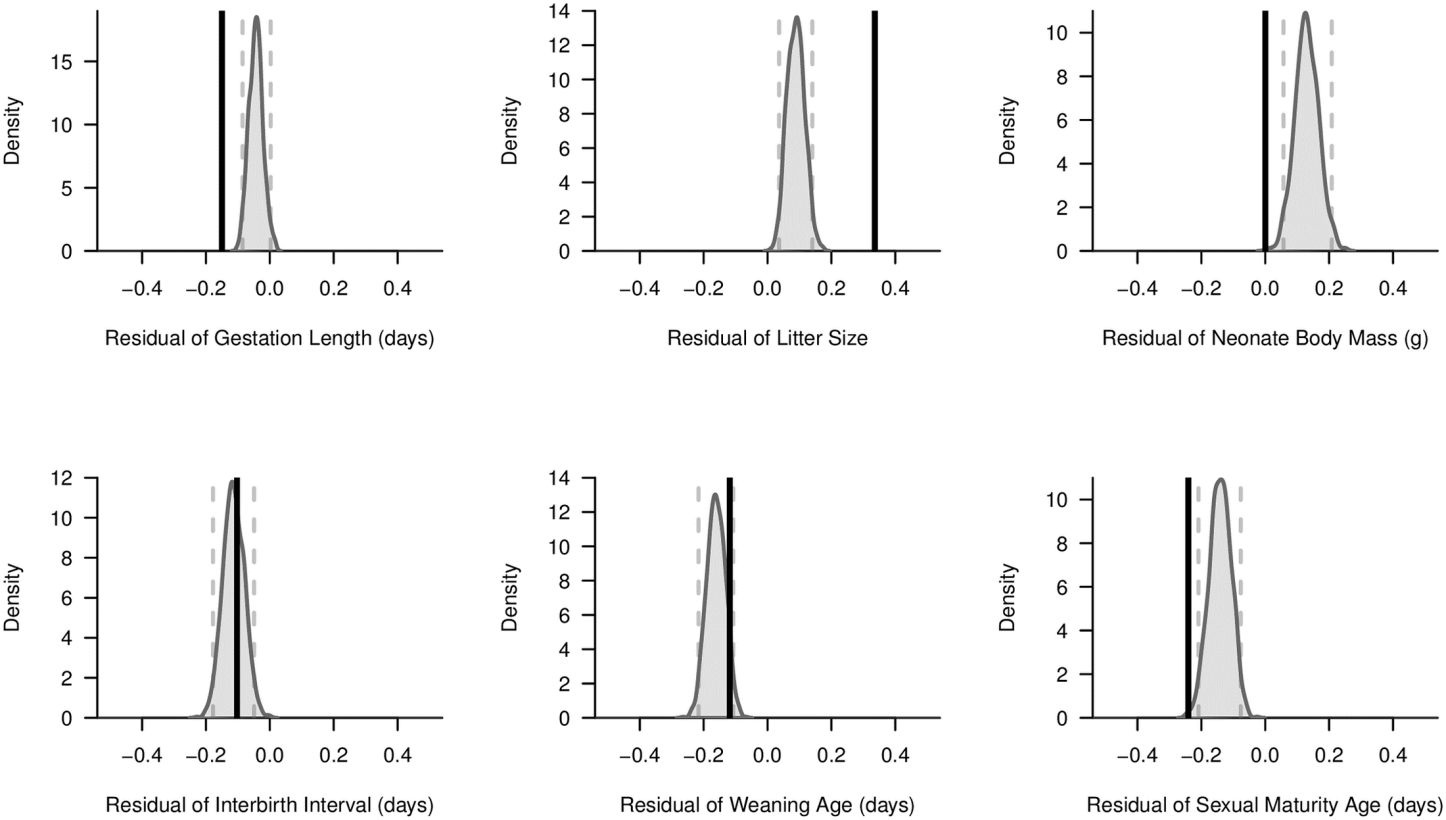
Kernel density plots comparing mean mass-corrected residual trait values for all mammalian species versus mammalian reservoir species. We conducted imputation tests using mass-corrected residual trait values that were generated by regressing six key life history traits on adult body mass (g). To generate an expected distribution of mean residual trait values, we randomly generated 1,000 sets of mammalian species that had the same taxonomic representation (at the order level) as the unique mammalian reservoir species we identified. By calculating the mean residual trait values for these random sets of species, we were able to generate the mean mass-corrected residual trait distributions displayed in grey. The 2.5^th^ and 97.5^th^ percentiles of each distribution are represented with dashed vertical grey lines. For comparison, the observed mean mass-corrected residual trait value of the identified mammalian reservoir species is shown as a vertical black line.

Using the imputed mammalian life history trait database, mammalian reservoir species were found to have extreme trait values relative to all mammals with respect to gestation length, litter size, neonate body mass, and age at sexual maturity (Fig 2). Reservoirs tended to have short gestation length, large litters, low neonate body mass, and a young age at sexual maturity. Reservoir mammals did not significantly differ from mammals as a whole with respect to interbirth interval and weaning age. A second analysis considering only reservoirs of human-target pathogens yielded very similar results (S1 Fig).

**Fig 3:**
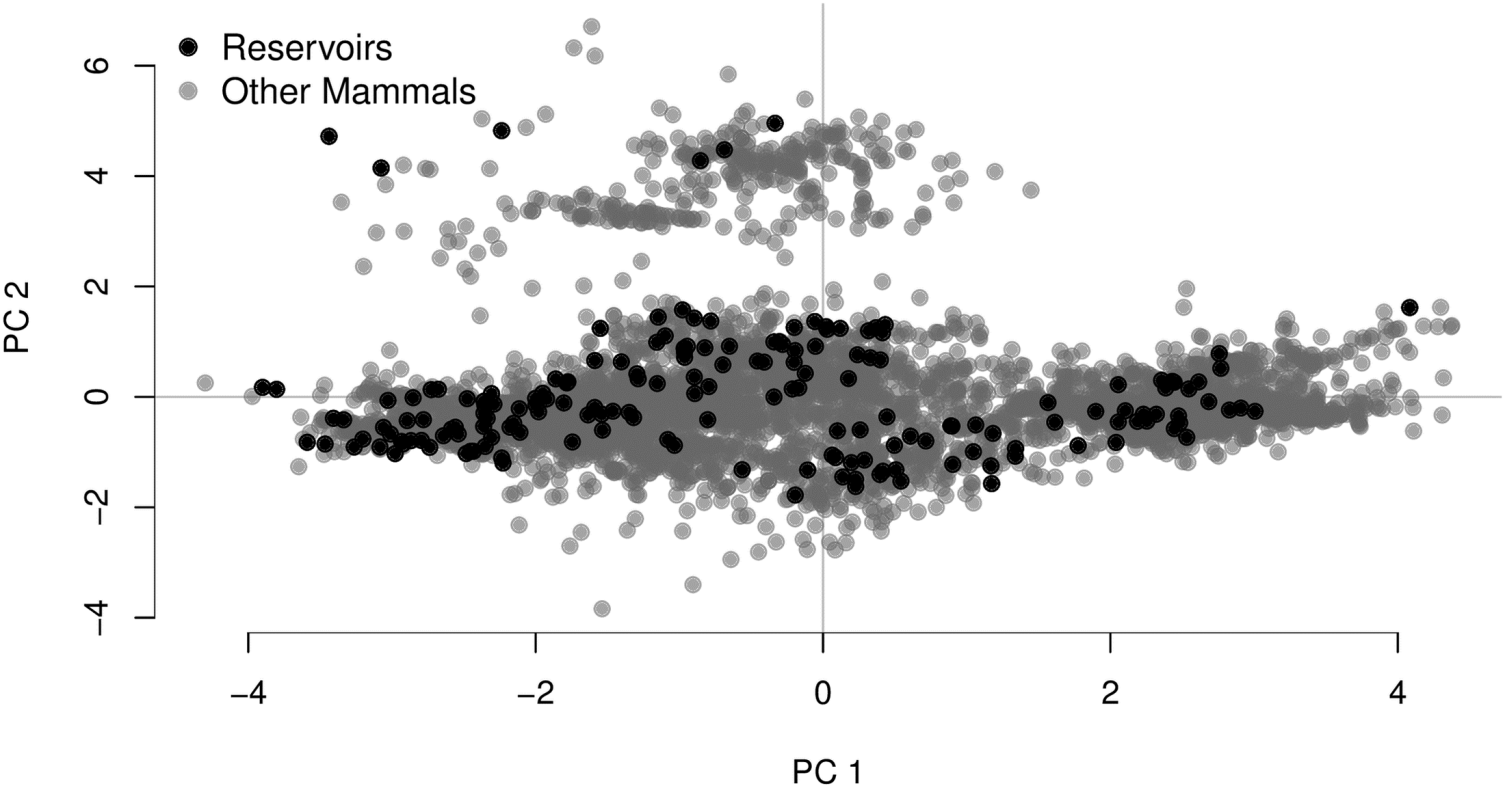
Principal component scores for mammalian species. We conducted principal components analysis using mass-corrected residual values for six key life history traits: gestation length (days), litter size, neonate body mass (g), interbirth interval (days), weaning age (days), and age at sexual maturity (days). Plotted in black are the first two PC scores for the 178 unique mammalian species we identified as reservoirs. Scores for all other mammal species are shown in grey.

**Fig 4:**
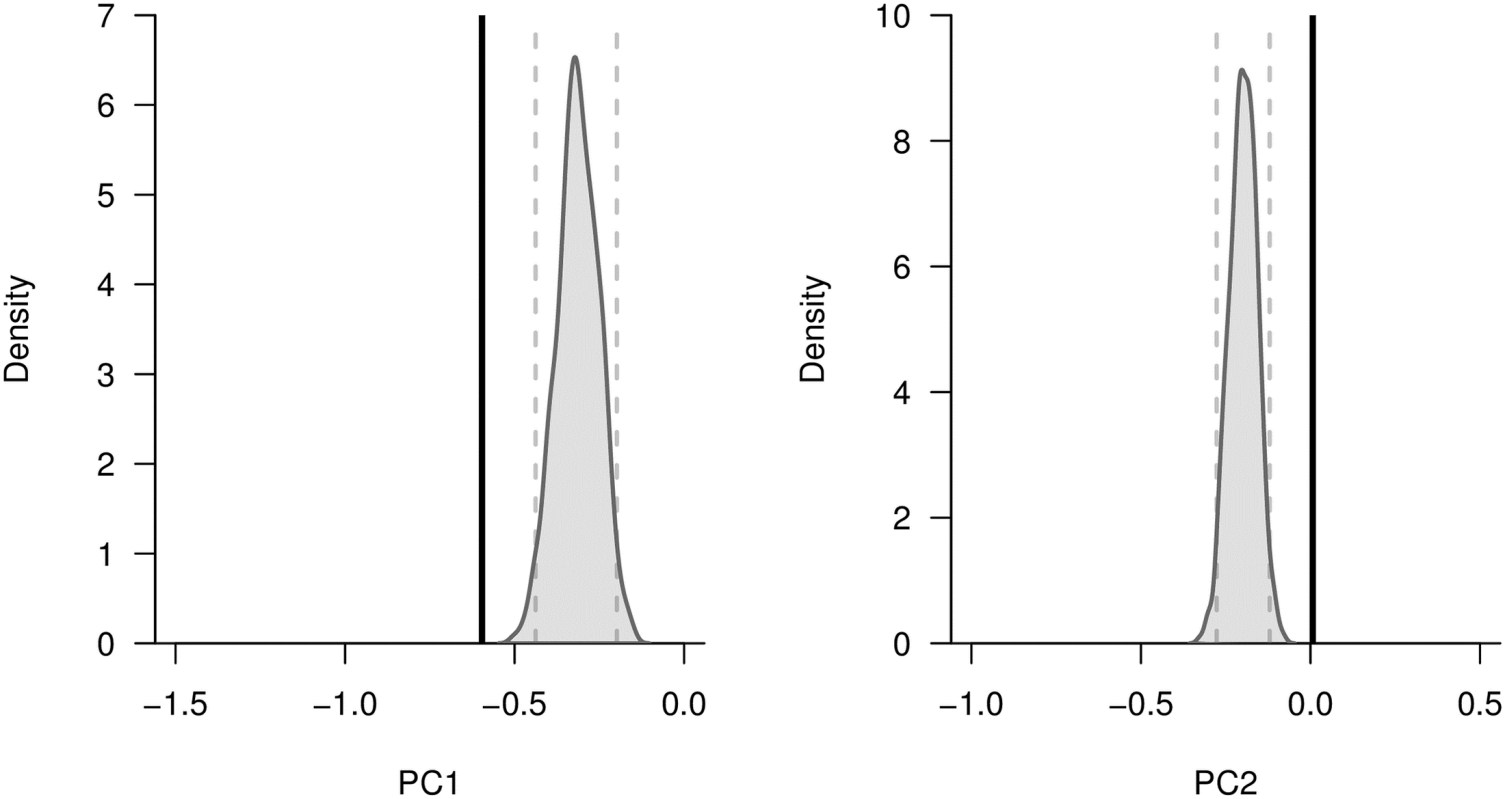
Kernel density plots comparing mean principal component scores for all mammalian species versus mammalian reservoir species. PCA was conducted on six key residual life history traits: gestation length (days), litter size, neonate body mass (g), interbirth interval (days), weaning age (days), and age at sexual maturity (days). We then used scores from the first two PC axes as variables of interest in imputation tests. To generate an expected distribution of mean PC scores, we randomly generated 1,000 sets of mammalian species that had the same taxonomic representation (at the order level) as the unique mammalian reservoir species we identified. By calculating the mean PC scores for these random sets of species, we were able to generate the mean PC score distributions displayed in grey. The 2.5^th^ and 97.5^th^ percentiles of each distribution are represented with dashed vertical grey lines. For comparison, the observed mean PC score of the identified mammalian reservoir species is shown as a vertical black line.

The first two principal component (PC) axes generated from our life history trait residual data together explained over 85% of the variance in the data, with PC1 accounting for 63% and PC2 for 23% (Table 6). The third PC axis only accounted for an additional 7% of the variance explained, and thus we restricted our analysis to the first two PC axes. The first PC axis contrasts litter size and the other life history traits: litter size loads negatively onto PC1 whereas the other life history traits have a positive association with that axis. Neonate body mass and gestation length both have relatively strong negative loadings on PC2, indicating that negative scores on this axis represent a longer gestation length that results in larger neonate body mass (Table 6). Permutation tests revealed that mammalian reservoir species are distinct from all mammals with respect to their PC scores: reservoirs have extreme negative scores for PC1 and extreme positive scores for PC2 (Figs 3 and 4).

**Table 6:** Loadings of mass-corrected life history trait residuals on the first two principal component axes.

	PC1	PC2
**Life History Traits and PC Loadings**		
Residual of gestation length (days)	0.45	-0.34
Residual of litter size	-0.42	0.20
Residual of neonate body mass (g)	0.20	-0.75
Residual of interbirth interval (days)	0.46	0.24
Residual of weaning age (days)	0.40	0.42
Residual of sexual maturity age (days)	0.46	0.23
**Proportion of Variance Explained**	0.63	0.23

Each of the six life history traits were regressed against adult body mass (g) to obtain mass-corrected residuals. The loadings of each trait residual variable on the first two principal components is given along with the proportion of variance explained by each component.

## Discussion

Pathogen spillover is a common phenomenon driving infectious disease in humans, domestic animals, and wildlife. Intuitively, one might expect that there are some features shared among reservoirs that not only enhance their ability to serve as hosts but also their tendency to be involved in pathogen spillover. While anecdotes and simple summaries indicate that rodents, bats, ungulates, and domestic animals are common sources of human disease, our analysis sought to include spillover pathogens affecting other animals as well. In addition, we aimed to account for publication biases and other attributes of the data that could skew inferences. Further, we investigated whether exceptional life history traits may underlie a mechanism by which reservoirs are associated with spillover. We find that most reservoirs of spillover pathogens include wild animals, include mammals, and are made up of a community. We found more artiodactyl and carnivore and fewer bat and primate species to be reservoir hosts than expected by chance. These findings may signify characteristics of these orders that predispose or prevent them from serving as reservoirs. High priority zoonotic pathogens are more likely to have avian reservoirs, while among pathogens with epidemic potential in the target population, primates are overrepresented despite being relatively uncommon reservoir hosts in general. Finally, compared to mammals overall, mammalian reservoir hosts tend to have faster mass-corrected life history characteristics, specifically those associated with high reproductive output rather than long-term survival.

There is widespread debate in the scientific literature surrounding the definition of a reservoir, with the term “reservoir” often being used loosely. Our study relied on a clearly-defined reservoir concept [6] to aid in interpretation of the scientific evidence for reservoir identity. For example, rather than simply equating natural infection with reservoir status, we sought evidence that would satisfy both parts of the Haydon et al. [6] definition. We made every effort to seek all available evidence for inconclusive cases but made some partially subjective decisions to exclude disease systems on the basis of insufficient data. We felt justified in these decisions since inclusion of disease systems based on weak or even incorrect data could bias our interpretations more than using a sparser (although possibly non-representative) dataset that included disease systems and host species that have received more extensive research attention.

Our analysis is partly premised upon the idea that the reservoirs are known for the disease systems we included, but undoubtedly there are many cases where more data would help inform our analyses. For example, one disease system that appears to involve a multiple species reservoir could actually include multiple cryptic disease cycles, each with only one reservoir host species. Indeed, multiple species may overlap in space and act as independent reservoirs without sharing the pathogen, as for cowpox virus in England where both bank voles (*Myodes glareolus*) and wood mice (*Apodemus sylvaticus*) serve as distinct, sympatric, single species reservoirs [47]. Alternatively, pathogens we report to have single species reservoirs may in fact depend on multiple species, some of which remain undocumented as hosts. Over half of the reservoirs in our database were classified as multiple species or complex, and two-thirds of disease systems were community-dependent. Our data therefore suggest that most spillover pathogens rely on a community rather than a single species, an idea recently proposed by Brisson et al. [48].

Rodents, artiodactyls, and carnivores predominate among reservoirs, likely due to a number of factors. First, rodents make up a large proportion (42%) of all mammal species. The rodent species that appear most often in reservoirs (*Rattus* spp. and *Mus musculus*) are associated with human-modified landscapes, and 90% of the disease systems included humans or a domestic animal as a target. Similarly, the association of artiodactyl livestock and domestic carnivores with humans likely drives their orders’ high representations among reservoirs, and some pathogens appear to have evolved to exploit the close contact between humans and livestock [49]. Known pathogens of livestock and domestic carnivores are often implicated in spillover events [2], and artificial selection for increased production in livestock can modify life history traits such that domestic species may be more likely to serve as pathogen reservoirs [50]. Finally, livestock species are kept in high densities, enabling rapid disease spread that can cause significant economic losses and attract increased attention from the scientific community.

Prior work suggested that carnivores (43.0%) and ungulates (Artiodactyla and Perissodactyla) (39.3%) are proportionally more prolific than rodents (22.5%) as hosts of human pathogens [2]. In contrast, we found that rodents are the most common mammalian reservoir hosts for zoonotic pathogens (Table 5). This discrepancy may be due to a difference in methodology. Reservoirs were designated as such in previous studies simply based on natural infection. For a given pathogen, evidence of natural infection in a dog, cat, or domestic ungulate is more likely than in other species, including wildlife, owing to the significant research attention given to domestic animals. In contrast, the present study used more stringent criteria for reservoir designation, which resulted in the identification of non-domestic taxa as important zoonotic pathogen reservoirs. However, the use of these criteria limited our sample size because hundreds of systems with weak evidence were excluded (S2 Dataset). This quality over quantity approach may variously bolster or weaken the conclusions presented here.

We hypothesized that the reservoir mammal species would differ from mammals as a whole with respect to their distribution among the 26 mammalian orders. Rodents were identified as an underrepresented order among all reservoirs but were overrepresented in the human-target disease system subset. In both cases, the magnitude of these differences was slight (Figs 1 and 2), and rodents were largely found within reservoirs at the same frequency as they are among all mammals. The underrepresentation of bats and shrews may be the result of their small size and difficulty of sampling. Using different methodology, Han et al. [51] largely found the same set of orders over- and underrepresented as reservoirs. For 11 of the 13 orders labeled in their Fig 2 [51], we find the same directional relationship (more species than expected or fewer species than expected). In contrast, we found both Diprotodontia and Primates to be underrepresented as reservoir hosts.

The overall distribution of reservoir mammals among orders did not significantly differ from the distribution observed among all known mammal species. This may reflect a propensity to study species of the most speciose orders. Han et al. [51] similarly found that the number of species in an order was predictive of the number of zoonotic hosts in the order. In contrast, the same analysis, but only considering human-target systems, found a significantly different distribution. This result suggests that mammalian reservoir hosts of human pathogens may be more and less likely to belong to the over- and underrepresented orders respectively, as presented here (S4 Table). Analysis at a finer phylogenetic scale may be able to identify families and genera that contain exceptionally high numbers of reservoirs.

While bats have been proposed as important reservoir hosts [8], they were largely underrepresented as reservoirs across all systems. One reason may be that bats are indeed a common reservoir, but that the evidence supporting them in that role was not strong enough to meet our confidence criteria (S2 Text). Evidence of natural infection in bats is established for dozens of viruses that infect humans [8], but our analysis only included eight with sufficient evidence in support of bats as the reservoir. Our study does support the role of bats as common reservoirs for high priority viruses that infect humans (Table 5, S6 and S7 Tables). This suggests that bats and potentially other taxa are particularly suited to act as reservoirs for certain types of pathogens. For example, the unique lifestyle and peculiarities of the immune system in bats may underlie their association with high-profile human viruses including Ebola, Hendra, Nipah, and SARS Coronavirus [10].

High priority zoonotic and epidemic potential pathogen reservoirs were more likely to include birds and primates than reservoirs overall. Birds are the source of high-profile food-borne pathogens (e.g., *Salmonella* spp. and *Campylobacter* spp.) and are implicated in recent emergence events for Influenza A virus and West Nile virus [52]. Non-human primates may serve as suitable reservoirs for epidemic disease in humans due to phylogenetic proximity and ease of cross-species pathogen transmission [53].

Understanding the influence of life history traits on infectious disease dynamics has been an active area of research. Host species with a high developmental tempo (i.e., short lifespans and large reproductive output) produce large numbers of susceptible individuals which can promote pathogen persistence and counteract short infective periods or the barrier of lifelong host immunity. The relationship between reservoir competence (defined as the product of a population’s susceptibility to infection and infectiousness to naïve vectors or hosts [11]) and pace of life has been investigated for a number of vector-borne pathogens. Studies have largely found support for a relationship between faster life history traits and greater reservoir competence [12,13]. For example, smaller-bodied *Borrelia burgdorferi* hosts and avian West Nile virus hosts with large clutch sizes were found to be more competent reservoir hosts [12].

Our results corroborate other findings that reservoir hosts tend to have fast life history traits. Our life history inferences are restricted to mammalian reservoirs as a consequence of data availability but agree well with a recent, comprehensive life history trait analysis of rodent reservoirs of human pathogens that identified early age at sexual maturity, short gestation length, and large litter size as among the strongest predictors of reservoir status [9].

Our analysis of life history traits in relation to reservoir status differed from past studies in two important ways. First, we examined mass-independent variation along the fast-slow life history continuum. Most life history traits are significantly correlated with body mass [34], with small species exhibiting greater reproductive output and faster developmental timing than larger species. By including all mammals listed in *Mammal Species of the World* [22], we covered a 10^8^-fold variation in mass between the smallest and largest species [31]. Controlling for mass, we found that the relationship between pace of life and reservoir status persisted. Second, we chose to impute missing values in an incomplete trait database rather than only analyzing species with complete trait data. Although imputing missing life history data leads to more accurate inference [39], collection of more empirical data on life history traits would be preferable. Life history traits may influence species’ abilities to occupy diverse environments. For example, birds in urbanized habitats appear to be especially fecund [54,55]. Traits associated with reservoir status (e.g., large litter size) may therefore characterize species that are also tolerant of anthropogenic habitat modification. The human population is growing, and disease emergence events have already been linked to anthropogenic disturbances [56]. If the same suite of life history traits predicts both reservoir status and urban adaptability, urban development may inadvertently select for biological communities enriched in pathogen reservoirs.

Identifying common patterns that characterize spillover pathogens and their reservoirs, such as those presented here, can help to develop useful frameworks for disease study and management [7,57]. Critically, our analysis focused on systems in which the reservoirs we identified are completely sufficient to maintain pathogens and are necessary for continued disease incidence in the target population. In such cases, management of the interspecies transmission interface and reservoir populations may prevent new cases. Diverse reservoir communities and their transmission dynamics may complicate disease management. For example, a transient or uncommon host species may act as a reservoir in the absence of a more permissive species [58]. Thus, control of the primary reservoir host may not effectively reduce disease burden and could, in fact, spread the pathogen to a new host. Effective management strategies therefore depend upon careful study of potential hidden reservoirs in disease systems of interest. We encourage future work that expands upon our database and seeks to identify new patterns to help understand infectious disease cycles.

## Supporting information

S1 Dataset Database of disease systems. (CSV)

S2 Dataset Database of excluded disease systems. The spreadsheet outlines the pathogen, pathogen type, target, region, reservoir, reservoir type, reservoir taxon, confidence of each considered, but excluded, system. The final column gives a reason for exclusion. While only one is listed, many systems met more than one criteria for exclusion.
(CSV)

S1 Text Column descriptions and references for S1 Dataset. (PDF)

S2 Text Criteria used to assign confidence levels to reservoir identities. (PDF)

S1 Fig Kernel density plots comparing mean mass-corrected residual trait values for all mammalian species versus mammalian reservoir species hosting pathogens with human targets. We conducted imputation tests using mass-corrected residual trait values that were generated by regressing six key life history traits on adult body mass (g). To generate an expected distribution of mean residual trait values, we randomly generated 1,000 sets of mammalian species that had the same taxonomic representation (at the order level) as the unique mammalian reservoir species we identified that hosted pathogens with human targets. By calculating the mean residual trait values for these random sets of species, we were able to generate the mean mass-corrected residual trait distributions displayed in grey. The 2.5^th^ and 97.5^th^ percentiles of each distribution are represented with dashed vertical grey lines. For comparison, the observed mean mass-corrected residual trait value of the identified mammalian reservoir species that host human pathogens is shown as a vertical black line.
(TIF)

S1 Table Geographical distribution of disease systems. Data show the percentage of disease systems found on each continent. In addition to the pathogen being endemic on a continent, the reservoir and target were also required to occur. Many systems (n = 99) were classified as worldwide and are thus present on each of the listed continents. The number of systems is given in parentheses. *The epidemic potential zone subsets represent the transmission potential of the pathogen in the target host population(s) following spillover. The zones are defined as follows: dead-end for a basic reproductive number (R_0_) nearly equal to zero, stuttering chains for an R_0_ greater than zero but less than one, and epidemic potential for an R_0_ greater than one. †High priority zoonotic pathogen subsets were determined by estimating the pathogen’s representation in the scientific literature using the H-index. Each subset was created to include pathogens that are among the 25% (Top 25%) and 10% (Top 10%) most significant known human pathogens.
(PDF)

S2 Table Summary of transmission types found in all disease systems and subsets. Data show the percentage of systems with each type of transmission between the reservoir and the target. A single system may be categorized as showing more than one transmission type. Trophic transmission included both food- and water-borne transmission. Vector-borne is limited to transmission by arthropods. The number of systems is given in parentheses. *The epidemic potential zone subsets represent the transmission potential of the pathogen in the target host population(s) following spillover. The zones are defined as follows: dead-end for a basic reproductive number (R_0_) nearly equal to zero, stuttering chains for an R_0_ greater than zero but less than one, and epidemic potential for an R_0_ greater than one. †High priority zoonotic pathogen subsets were determined by estimating the pathogen’s representation in the scientific literature using the H-index. Each subset was created to include pathogens that are among the 25% (Top 25%) and 10% (Top 10%) most significant known human pathogens.
(PDF)

S3 Table Observed representation of mammalian orders among reservoirs versus expected. The expected distribution represents the number of species in each order that would be identified as reservoirs if they were found as a reservoir host in the same proportions as they are all mammals. Observed numbers total to the 178 mammalian species identified as reservoir hosts. The residual is the absolute difference between expected species and observed species. Standardized residuals are calculated by dividing the residual by the square root of the expected number of species. Positive residuals represent more reservoir species than expected by chance. Negative residuals represent fewer reservoir species than expected by chance. *As listed by *Mammal Species of the World* [22].
(PDF)

S4 Table Observed representation of mammalian orders among reservoirs versus expected for human target systems. The expected distribution represents the number of species in each order that would be identified as reservoirs if they were found as a reservoir host of a human-target pathogen in the same proportions as they are all mammals. Observed numbers total to the 155 mammalian species identified as reservoir hosts. The residual is the absolute difference between expected species and observed species. Standardized residuals are calculated by dividing the residual by the square root of the expected number of species. Positive residuals represent more reservoir species than expected by chance. Negative residuals represent fewer reservoir species than expected by chance. *As listed by *Mammal Species of the World* [22].
(PDF)

S5 Table Most represented mammal species among reservoirs. (PDF)

S6 Table Summary of mammalian reservoirs by orders represented in subsets of viral pathogen systems and viral pathogen systems with human targets. Each reservoir was categorized by the orders of mammals represented by the species it included. Orders reflect the hierarchy detailed in *Mammal Species of the World* [22]. The data above show the percentage of systems whose reservoirs include at least one species belonging to the order. The number of systems is given in parentheses. Unlisted mammalian orders were not found among reservoirs. The ‘All Systems’ and ‘Human Target’ columns are redundant with Table 5 and included here for comparison.
(PDF)

S7 Table Observed representation of unique reservoir species in mammalian orders versus expected for subsets of viral pathogen systems (A) and viral pathogen systems that include humans as a target (B). The expected distribution represents the number of species in each order that would be identified as reservoirs if they were found as a reservoir host in the same proportions as they are all mammals. Observed numbers are based on the number of unique mammalian species identified as reservoir hosts in each subset (92 in A; 80 in B). Only orders with observed reservoir species in the viral pathogen subset are shown. The residual is the absolute difference between expected species and observed species. Standardized residuals are calculated by dividing the residual by the square root of the expected number of species. Positive residuals represent more reservoir species than expected by chance. Negative residuals represent fewer reservoir species than expected by chance. *As listed by *Mammal Species of the World* [22].
(PDF)
