# Supplementary material for: Are disease reservoirs special? Taxonomic and life history characteristics
Source: PLoS One. 2017 Jul 13;12(7):e0180716. doi: 10.1371/journal.pone.0180716 (PMC5509157; doi:10.1371/journal.pone.0180716)
Supplement: S1 Text — (PDF) [file pone.0180716.s003.pdf]

S1 Text. Column descriptions and references for S1 Dataset.

Contents

I. Column Descriptions
II. References

I. Column Descriptions

Bold type references column of that name.

**Column Header**	**Description**
id	A unique identifier for each system.
pathogen	Scientific name of the pathogen species, strain, or type.
type	The major group of the **pathogen**. Limited to arthropod, bacteria, fungus, helminth, oomycete, protozoa, or virus.
target	The species to which the **pathogen** spills over from the **reservoir**. Listed as common species or group names only.
human.target	A binary variable. ‘1’ if humans are included as a **target**, ‘0’ if they are not.
domestic.target	A binary variable. ‘1’ if domestic animals are included as a **target**, ‘0’ if they are not. Because many species listed as targets exist in both domestic and wild populations, we determined specifically if the domestic populations are a target for spillover.
wild.target	A binary variable. ‘1’ if wild animals are included as a **target**, ‘0’ if they are not. Because many species listed as targets exist in both wild and domestic populations, we determined specifically if the wild populations are a target for spillover.
livestock.target	A binary variable. ‘1’ if a livestock species is included as a **target**, ‘0’ if none are. Because many species listed as targets exist in both wild and domestic livestock populations, we determined specifically if the domestic livestock populations are a target for spillover.
companion.target	A binary variable. ‘1’ if a companion animal species is included as a **target**, ‘0’ if none are. All target species identified as companion animals were either domestic dogs or domestic cats.
transmission	Specifically, how the **pathogen** is transmitted from the **reservoir** to the **target**. Standardized to one or more of direct contact (DC), indirect contact (IC), trophic (T, including food- and water-borne transmission), and vector-borne (VB, specifically by biological arthropod vectors).
direct	A binary variable describing whether or not direct contact transmission is reported from the **reservoir** to the **target**.
indirect	A binary variable describing whether or not indirect contact transmission is reported from the **reservoir** to the **target**.
vector	A binary variable describing whether or not vector-borne transmission is reported from the **reservoir** to the **target**.
trophic	A binary variable describing whether or not trophic transmission is reported from the **reservoir** to the **target**.

vector.id	If **vector** is '1', the common name(s) of arthropod vector group(s) responsible for transmission from the **reservoir** to the **target**.
fleas	A binary variable describing whether or not fleas are reported as a biological vector from the **reservoir** to the **target**.
ticks	A binary variable describing whether or not ticks are reported as a biological vector from the **reservoir** to the **target**.
mosquitoes	A binary variable describing whether or not mosquitoes are reported as a biological vector from the **reservoir** to the **target**.
flies	A binary variable describing whether or not flies are reported as a biological vector from the **reservoir** to the **target**.
vector.req	Answers the question, *if vector-borne transmission is reported from the* ***reservoir*** *to the* ***target****, is the presence of the vector required for continued spillover?*
region	All regions and continents where the described spillover between **reservoir** and **target** are known to occur. Worldwide used if the system is reported on six continents.
region.standard	Region standardized to continent and worldwide abbreviations. Limited to worldwide (W), Africa (AF), Asia (AS), Australia/Oceania (AU), Europe (EU), North America (NA), and South America (SA).
n.america	A binary variable describing whether or not North America is reported as a location of spillover. '0' is used when region is listed as worldwide.
s.america	A binary variable describing whether or not South America is reported as a location of spillover. '0' is used when region is described as worldwide.
europe	A binary variable describing whether or not Europe is reported as a location of spillover. '0' is used when region is described as worldwide.
asia	A binary variable describing whether or not Asia is reported as a location of spillover. '0' is used when region is described as worldwide.
africa	A binary variable describing whether or not Africa is reported as a location of spillover. '0' is used when region is described as worldwide.
oceania	A binary variable describing whether or not Oceania is reported as a location of spillover. '0' is used when region is described as worldwide.
worldwide	A binary variable describing whether or not worldwide is used to describe the locations of spillover. Systems were classified as worldwide if the disease system described is known to occur on six continents.
reservoir	The populations and environments that maintain the **pathogen** and serve as the source of new cases in the **target** population(s). Based on the definition given in Haydon et al. (2002).
environment	A binary variable describing whether or not an abiotic environment is included in the **reservoir**.

domestic.reservoir	A binary variable describing whether or not the **reservoir** includes domestic animals. Because species listed within the **reservoir** often exist in both domestic and wild populations, we determined specifically if domestic populations are a source for spillover.
wild.reservoir	A binary variable describing whether or not the **reservoir** includes wild animals. Because species listed within the **reservoir** often exist in both wild and domestic populations, we determined specifically if wild populations are a source for spillover.
livestock.reservoir	A binary variable describing whether or not the **reservoir** includes livestock. Because species listed within the **reservoir** often exist in both domestic livestock and wild populations, we determined specifically if livestock populations are a source for spillover.
companion.reservoir	A binary variable describing whether or not the **reservoir** includes companion animals. Because species listed within the **reservoir** often exist as both companion animals and in wild populations, we determined specifically if companion animal populations are a source for spillover.
reservoir.type	Describes each **reservoir** as single species, multiple species, or complex. Single species is used for systems where exactly one species, identified to the species level, is listed in the **reservoir** field. Multiple species is used for systems that both have more than one species listed in the **reservoir** field and where there is no defined cycle among the multiple species. Complex is used for systems where the pathogen completes a single generation across more than one host or environment. In the **reservoir** field, complex reservoirs are written as a cycle with the hosts or environments at each stage separated by a '+'. For example, *environment + first intermediate host + second intermediate host + definitive host*.
single	A binary variable describing whether or not the **reservoir** is categorized as single species.
multiple	A binary variable describing whether or not the **reservoir** is categorized as multiple species.
complex	A binary variable describing whether or not the **reservoir** is categorized as complex.
taxon	A comma-separated list of the major animal taxa represented in each **reservoir**. Limited to: amphibians, annelids, arthropods, birds, fish, helminths, mammals, mollusks, reptiles.
mammal	A binary variable describing whether or not mammals are included in the **reservoir**.
arthropod	A binary variable describing whether or not arthropods are included in the **reservoir**. If an arthropod vector is listed in **vector** but not in the **reservoir** field this field was set to '0'.
fish	A binary variable describing whether or not fish are included in the **reservoir**.
amphibian	A binary variable describing whether or not amphibians are included in the **reservoir**.

bird	A binary variable describing whether or not birds are included in the **reservoir**.
annelid	A binary variable describing whether or not annelids are included in the **reservoir**.
helminth	A binary variable describing whether or not helminths are included in the **reservoir**.
reptile	A binary variable describing whether or not reptiles are included in the **reservoir**.
order	A comma-separated list of the mammalian orders represented in the **reservoir**. Limited to the following orders that appeared in this database: Artiodactyla, Carnivora, Cetacea, Chiroptera, Cingulata, Didelphimorphia, Diprotodontia, Erinaceomorpha, Hyracoidea, Lagomorpha, Perissodactyla, Pilosa, Primates, Rodentia, Soricomorpha.
artiodactyla	A binary variable describing whether or not a member of the order Artiodactyla is included in the **reservoir**.
carnivora	A binary variable describing whether or not a member of the order Carnivora is included in the **reservoir**.
rodentia	A binary variable describing whether or not a member of the order Rodentia is included in the **reservoir**.
perissodactyla	A binary variable describing whether or not a member of the order Perissodactyla is included in the **reservoir**.
primates	A binary variable describing whether or not a member of the order Primates is included in the **reservoir**.
cetacea	A binary variable describing whether or not a member of the order Cetacea is included in the **reservoir**.
soricomorpha	A binary variable describing whether or not a member of the order Soricomorpha is included in the **reservoir**.
chiroptera	A binary variable describing whether or not a member of the order Chiroptera is included in the **reservoir**.
cingulata	A binary variable describing whether or not a member of the order Cingulata is included in the **reservoir**.
didelphimorphia	A binary variable describing whether or not a member of the order Didelphimorphia is included in the **reservoir**.
diprotodontia	A binary variable describing whether or not a member of the order Diprotodontia is included in the **reservoir**.
erinaceomorpha	A binary variable describing whether or not a member of the order Erinaceomorpha is included in the **reservoir**.
hyracoidea	A binary variable describing whether or not a member of the order Hyracoidea is included in the **reservoir**.
lagomorpha	A binary variable describing whether or not a member of the order Lagomorpha is included in the **reservoir**.
pilosa	A binary variable describing whether or not a member of the order Pilosa is included in the **reservoir**.

confidence	The confidence level (See S2 Text) assigned to the system. Limited to medium and high because systems with confidence of low and none were excluded from this database.
rozone	Epidemic potential zone of the system describing the transmission from **reservoir** to **target**. The possible entries are: D (dead-end) for an R_0 equal to zero, S (stuttering chains) for an R_0 greater than zero but less than one, and E (epidemic potential) for an R_0 greater than one.
hindex	The H index of the **pathogen** as calculated by Web of Science in September 2015. Only applies to systems with humans listed as a **target**. All others are 'NA'.
top10	A binary variable describing whether or not the system is included in the top 10% high priority zoonotic pathogen (HPZP) subset. Is '1' where **hindex** is greater than 102.7.
top25	A binary variable describing whether or not the system is included in the top 25% high priority zoonotic pathogen (HPZP) subset. Is '1' where **hindex** is greater than 55.
references	Sources of evidence to support the **reservoir** listed. See Section II of this file for citations.

II. References

1. Thompson, G.M., S. Jess, and A.K. Murchie, *A review of African horse sickness and its implications for Ireland.* Irish Veterinary Journal, 2012. **65**: p. e9.
2. Spickler, A.R. *African Horse Sickness*. 2015 February 2015 [cited 2015; Available from: http://www.cfsph.iastate.edu/Factsheets/pdfs/african_horse_sickness.pdf.
3. Jori, F. and A.D.S. Bastos, *Role of wild suids in the epidemiology of African swine fever.* EcoHealth, 2009. **6**: p. 296-310.
4. Sood, R., et al., *Detection and molecular characterization of naturally transmitted sheep associated malignant catarrhal fever in cattle in India.* Tropical Animal Health and Production, 2014. **46**(6): p. 1037-1043.
5. Russell, G.C., J.P. Stewart, and D.M. Haig, *Malignant catarrhal fever: a review.* The Veterinary Journal, 2009. **179**: p. 324-335.
6. Li, H., et al., *Malignant catarrhal fever: Inching toward understanding.* Annual Review of Animal Biosciences, 2014. **2**: p. 209-233.
7. Dodd, K.A., et al., *Kyasanur forest disease virus infection in mice is associated with higher morbidity and mortality than infection with the closely related Alkhurma hemorrhagic fever virus.* PLOS One, 2014. **9**(6): p. e100301.
8. Alzahrani, A.G., et al., *Alkhurma hemorrhagic fever in humans, Najran, Saudi Arabia.* Emerging Infectious Diseases, 2010. **16**(12): p. 1882-1888.
9. Ma, C., et al., *Hantaviruses in rodents and humans, Xi'an, PR China.* Journal of General Virology, 2012. **93**(10): p. 2227-2236.
10. Foley, J.E., et al., *Anaplasma phagocytophilum infection in small mammal hosts of Ixodes ticks, western United States.* Emerging Infectious Diseases, 2008. **14**(7): p. 1147-1150.
11. Keesing, F., et al., *Prevalence of human-active and variant 1 strains of the tick-borne pathogen Anaplasma phagocytophilum in hosts and forests of Eastern North America.* The American Journal of Tropical Medicine and Hygiene, 2014. **91**(2): p. 302-309.
12. Bown, K.J., et al., *Seasonal dynamics of Anaplasma phagocytophila in a rodent-tick (Ixodes trianguliceps) system, United Kingdom.* Emerging Infectious Diseases, 2003. **9**(1): p. 63-70.
13. Bowman, D.D., et al., *Hookworms of dogs and cats as agents of cutaneous larva migrans.* Trends in Parasitology, 2010. **26**(4): p. 162-167.
14. Traub, R.J., et al., *PCR-based coprodiagnostic tools reveal dogs as reservoirs of zoonotic anclyostomiasis caused by Anclyostoma ceylanicum in temple communities in Bangkok.* Veterinary Parasitology, 2008. **155**: p. 67-78.
15. Traub, R.J., *Anclyostoma ceylanicum-a re-emerging but neglected parasitic zoonosis.* International Journal of Parasitology, 2013. **43**: p. 1009-1013.
16. Schmaljohn, C. and B. Hjelle, *Hantaviruses: a global disease problem.* Emerging Infectious Diseases, 1997. **3**(2): p. 95-104.
17. Romero-Algeria, A., et al., *Angiostrongylus costaricensis: Systematic review of case reports.* Advances in Infectious Diseases, 2014. **4**: p. 36-41.
18. Morgan, E.R., et al., *Angiostrongylus vasorum: a real heartbreaker.* TRENDS in Parasitology, 2005. **21**(1): p. 49-51.
19. Audicana, M.T. and M.W. Kennedy, *Anisakis simplex: from obscure infectious worm to inducer of immune hypersensitivity.* Clinical Microbiology Reviews, 2008. **21**(2): p. 360-379.

20. Aquiloni, L., et al., *The north American crayfish Procambarus clarkii is the carrier of the oomycete Aphanomyces astaci in Italy.* Biological Invasions, 2011. **13**: p. 359-367.
21. Limongi, J.E., et al., *Serological survey of hantavirus in rodents in Uberlândia, Minas Gerais, Brazil.* Revista do Instituto de Medicina Tropical de São Paulo, 2013. **55**(3): p. 155-158.
22. de Araujo, J., et al., *Detection of hantavirus in bats from remaining rain forest in São Paulo, Brazil.* BMC research notes, 2012. **5**(1): p. 690.
23. De Sousa, R.L.M., et al., *Natural host relationships and genetic diversity of rodent-associated hantaviruses in southeastern Brazil.* Intervirology, 2008. **51**(4): p. 299-310.
24. Suzuki, A., et al., *Identifying rodent hantavirus reservoirs, Brazil.* Emerging Infectious Diseases, 2004. **10**: p. 2127-2134.
25. Drabick, J.J., *Pentastomiasis.* Review of Infectious Diseases, 1987. **9**(6): p. 1087-1094.
26. *Pentastomiasis*. 2008 May 19, 2008 [cited 2015.
27. Slifko, T.R., H.V. Smith, and J.B. Rose, *Emerging parasite zoonoses associated with water and food.* International Journal for Parasitology, 2000. **30**(12): p. 1379-1393.
28. Tantrawatpan, C., et al., *Genetic differentiation between Artyfechinostomum malayanum and A. sufrartyfex (Tremotoma: Echinostomatidae) based on internal transcribed spacer sequences.* Parasitology Research, 2013. **112**: p. 437-441.
29. Santos, C.P., et al., *Fish-borne trematodosis: Potential risk of infection of Ascocotyle (Phagicola) longa (Heterophyidae).* Veterinary Parasitology, 2013. **193**: p. 302-306.
30. Simoes, S.B.E., H.S. Barbosa, and C.P. Santos, *The life cycle of Ascocotyle (Phagicola) longa (Digenea: Heterophyidae): a causative agent of fish-borne trematodosis.* Acta Tropica, 2010. **113**: p. 226-233.
31. Guyatt, K.J., et al., *A molecular epidemiological study of Australian bat lyssavirus.* Journal of General Virology, 2003. **84**: p. 485-496.
32. Hanna, J.N., et al., *Australian bat lyssavirus infection: a second human case, with a long incubation period.* The Medical Journal of Australia, 2000. **172**(2): p. 597-599.
33. Galuppi, R., et al., *Detection and molecular characterization of Theileria sp. in fallow deer (Dama dama) and ticks from an Italian natural preserve.* Research in Veterinary Science, 2011. **91**(1): p. 110-115.
34. Michel, A.O., A. Mathis, and M.-P. Ryser-Degiorgis, *Babesia spp. in European wild ruminant species: parasite diversity and risk factors for infection.* Veterinary Research, 2014. **45**(1): p. e65.
35. Hersh, M.H., et al., *Reservoir competence of wildlife host species for Babesia microti.* Emerging Infectious Diseases, 2012. **18**(12): p. 1951-1957.
36. Zamoto-Niikura, A., et al., *Sika deer carrying Babesia parasites closely related to B. divergens, Japan.* Emerging infectious diseases, 2014. **20**(8): p. 1398-1400.
37. Yeagley, T.J., et al., *Detection of Babesia gibsoni and the canine small Babesia 'Spanish isolate' in blood samples obtained from dogs confiscated from dogfighting operations.* Journal of the American Veterinary Medical Association, 2009. **235**(5): p. 535-539.
38. Baneth, G., et al., *Reclassification of Theileria annae as Babesia vulpes sp. nov.* Parasites and Vectors, 2015. **8**(1): p. e207.
39. Najm, N.-A., et al., *A molecular survey of Babesia spp. and Theileria spp. in red foxes (Vulpes vulpes) and their ticks from Thuringia, Germany.* Ticks and Tick-borne Diseases, 2014. **5**(4): p. 386-391.

40. Ganz, H.H., et al., *Interactions between Bacillus anthracis and plants may promote anthrax transmission.* PLOS: Neglected Tropical Diseases, 2014. **8**(6): p. e2903.
41. Ajeagah, G.A. and Y. Moussima, *Study of the influence of environmental factors on the occurrence of Balantidium coli cysts in an urban aquatic system in Cameroon.* Journal of Ecology and the Natural Environment, 2014. **6**(6): p. 190-199.
42. Heller, R., et al., *Bartonella alsatica sp. nov., a new Bartonella species isolated from the blood of wild rabbits.* International Journal of Systematic Bacteriology, 1999. **49**(1): p. 283-288.
43. Heller, R., et al., *Prevalence of Bartonella henselae and Bartonella clarridgeiae in stray cats.* Journal of Clinical Microbiology, 1997. **35**(6): p. 1327-1331.
44. Ellis, B., et al., *Rats of the genus Rattus are reservoir hosts for pathogenic Bartonella species: an Old World origin for a New World disease?* Journal of Infectious Diseases, 1999. **180**(1): p. 220-224.
45. Holmberg, M., et al., *Bartonella infection in sylvatic small mammals of central Sweden.* Epidemiology and Infection, 2003. **130**(1): p. 149-157.
46. Jardine, C., et al., *Rodent-associated Bartonella in Saskatchewan, Canada.* Vector-Borne and Zoonotic Diseases, 2005. **5**(4): p. 402-409.
47. Kordick, D.L., et al., *Clinical and pathologic evaluation of chronic Bartonella henselae or Bartonella clarridgeiae infection in cats.* Journal of Clinical Microbiology, 1999. **37**(5): p. 1536-1547.
48. Avidor, B., et al., *Bartonella koehlerae, a new cat-associated agent of culture-negative human endocarditis.* Journal of Clinical Microbiology, 2004. **42**(8): p. 3462-3468.
49. Matsumoto, K., et al., *Molecular detection of Bartonella schoenbuchensis from ectoparasites of deer in Massachusetts.* Vector-borne and Zoonotic Diseases, 2008. **8**(4): p. 549-554.
50. Bai, Y., et al., *Bartonella vinsonii subsp. arupensis in humans, Thailand.* Emerging Infectious Diseases, 2012. **18**(6): p. 989-991.
51. Chang, C.-C., et al., *Seroepidemiology of Bartonella vinsonii subsp. berkhoffii infection in California coyotes, 1994-1998.* Emerging Infectious Diseases, 1999. **5**(5): p. 711-715.
52. Weldon, C., et al., *Origin of the amphibian chytrid fungus.* Emerging Infectious Diseases, 2004. **10**(12): p. 2100-2105.
53. Soto-Azat, C., et al., *Widespread historical presence of Batrachochytrium dendrobatidis in African pipid frogs.* Diversity and Distributions, 2010. **16**(1): p. 126-131.
54. Daszak, P., et al., *Experimental evidence that the bullfrog (Rana catesbeiana) is a potential carrier of chytridiomycosis, an emerging fungal disease of amphibians.* Herpetological Journal, 2004. **14**(4): p. 201-207.
55. Hanselmann, R., et al., *Presence of an emerging pathogen of amphibians in introduced bullfrogs Rana catesbeiana in Venezuela.* Biological Conservation, 2004. **120**(1): p. 115-119.
56. Schloegel, L., et al., *The North American bullfrog as a reservoir for the spread of Batrachochytrium dendrobatidis in Brazil.* Animal Conservation, 2010. **13**(1): p. 53-61.
57. Reeder, N.M., A.P. Pessier, and V.T. Vredenburg, *A reservoir species for the emerging amphibian pathogen Batrachochytrium dendrobatidis thrives in a landscape decimated by disease.* PLoS One, 2012. **7**(3): p. e33567.

58. Wise, M.E., et al., *Severe and fatal central nervous system disease in humans caused by Baylisascaris procyonis, the common roundworm of raccoons: a review of current literature.* Microbes and Infection, 2005. **7**(2): p. 317-323.
59. McIntyre, N.E., et al., *A longitudinal study of Bayou virus, hosts, and habitat.* The American Journal of Tropical Medicine and Hygiene, 2005. **73**(6): p. 1043-1049.
60. Padula, P., et al., *Andes virus and first case report of Bermejo virus causing fatal pulmonary syndrome.* Emerging Infectious Diseases, 2002. **8**(4): p. 437-439.
61. Rivera, P.C., et al., *Molecular identification and phylogenetic relationships among the species of the genus Oligoryzomys (Rodentia, Cricetidae) present in Argentina, putative reservoirs of hantaviruses.* Zoologica Scripta, 2007. **36**(3): p. 231-239.
62. González-Ittig, R.E., et al., *The molecular phylogenetics of the genus Oligoryzomys (Rodentia: Cricetidae) clarifies rodent host–hantavirus associations.* Zoological Journal of the Linnean Society, 2014. **171**(2): p. 457-474.
63. Denegri, G.M. and J. Perez-Serrano, *Bertiellosis in man: A review of cases.* Revista do Instituto de Medicina Tropical de São Paulo, 1997. **39**: p. 123-128.
64. Glass, G.E., et al., *Black Creek Canal virus infection in Sigmodon hispidus in southern Florida.* The American Journal of Tropical Medicine and Hygiene, 1998. **59**(5): p. 699-703.
65. Hutchinson, K.L., et al., *Transmission of Black Creek Canal virus between cotton rats.* Journal of Medical Virology, 2000. **60**(1): p. 70-76.
66. Mehlhorn, H., et al., *First occurrence of Culicoides obsoletus-transmitted Bluetongue virus epidemic in Central Europe.* Parasitology Research, 2007. **101**(1): p. 219-228.
67. Lynch, S., et al., *Observations raise the question if the Pacific oyster, Crassostrea gigas, can act as either a carrier or a reservoir for Bonamia ostreae or Bonamia exitiosa.* Parasitology, 2010. **137**(10): p. 1515-1526.
68. Hanincova, K., et al., *Association of Borrelia afzelii with rodents in Europe.* Parasitology, 2003. **126**(1): p. 11-20.
69. Humair, P., O. Rais, and L. Gern, *Transmission of Borrelia afzelii from Apodemus mice and Clethrionomys voles to Ixodes ricinus ticks: differential transmission pattern and overwintering maintenance.* Parasitology, 1999. **118**(1): p. 33-42.
70. Skuballa, J., et al., *Occurrence of different Borrelia burgdorferi sensu lato genospecies including B. afzelii, B. bavariensis, and B. spielmanii in hedgehogs (Erinaceus spp.) in Europe.* Ticks and Tick-borne Diseases, 2012. **3**(1): p. 8-13.
71. Huegli, D., et al., *Apodemus species mice are reservoir hosts of Borrelia garinii OspA serotype 4 in Switzerland.* Journal of Clinical Microbiology, 2002. **40**(12): p. 4735-4737.
72. Lane, R., et al., *Refractoriness of the western fence lizard (Sceloporus occidentalis) to the Lyme disease group spirochete Borrelia bissettii.* Journal of Parasitology, 2006. **92**(4): p. 691-696.
73. Fedorova, N., et al., *Remarkable diversity of tick or mammalian-associated Borreliae in the metropolitan San Francisco Bay Area, California.* Ticks and Tick-Borne Diseases, 2014. **5**(6): p. 951-961.
74. Brunner, J.L., K. LoGiudice, and R.S. Ostfeld, *Estimating reservoir competence of Borrelia burgdorferi hosts: prevalence and infectivity, sensitivity, and specificity.* Journal of Medical Entomology, 2008. **45**(1): p. 139-147.
75. Anderson, J.M. and D.E. Norris, *Genetic diversity of Borrelia burgdorferi sensu stricto in Peromyscus leucopus, the primary reservoir of Lyme disease in a region of endemicity in*

southern Maryland. Applied and Environmental Microbiology, 2006. **72**(8): p. 5331-5341.
76. Lane, R.S., et al., *Western gray squirrel (Rodentia: Sciuridae): a primary reservoir host of Borrelia burgdorferi in Californian oak woodlands?* Journal of Medical Entomology, 2005. **42**(3): p. 388-396.
77. Salkeld, D.J., et al., *Seasonal activity patterns of the western black-legged tick, Ixodes pacificus, in relation to onset of human Lyme disease in northwestern California.* Ticks and Tick-borne Diseases, 2014. **5**(6): p. 790-796.
78. Pisanu, B., et al., *High prevalence of Borrelia burgdorferi sl in the European red squirrel Sciurus vulgaris in France.* Ticks and Tick-borne Diseases, 2014. **5**(1): p. 1-6.
79. Taragel'ová, V., et al., *Blackbirds and song thrushes constitute a key reservoir of Borrelia garinii, the causative agent of borreliosis in Central Europe.* Applied and Environmental Microbiology, 2008. **74**(4): p. 1289-1293.
80. Schwan, T.G., et al., *Diversity and distribution of Borrelia hermsii.* Emerging Infectious Diseases, 2007. **13**(3): p. 436-442.
81. Barbour, A.G., et al., *Niche partitioning of Borrelia burgdorferi and Borrelia miyamotoi in the same tick vector and mammalian reservoir species.* The American Journal of Tropical Medicine and Hygiene, 2009. **81**(6): p. 1120-1131.
82. Barbour, A.G., et al., *Niche partitioning of Borrelia burgdorferi and Borrelia miyamotoi in the same tick vector and mammalian reservoir species.* The American Journal of Tropical Medicine and Hygiene, 2009. **81**(6): p. 1120-1131.
83. Richter, D., et al., *Relationships of a novel Lyme disease spirochete, Borrelia spielmani sp. nov., with its hosts in Central Europe.* Applied and Environmental Microbiology, 2004. **70**(11): p. 6414-6419.
84. Meagher, M. and M.E. Meyer, *On the origin of brucellosis in bison of Yellowstone National Park: a review.* Conservation Biology, 1994. **8**(3): p. 645-653.
85. Meyer, M.E. and M. Meagher, *Brucellosis in free-ranging bison (Bison bison) in Yellowstone, Grand Teton, and Wood Buffalo National Parks: a review.* Journal of Wildlife Diseases, 1995. **31**(4): p. 579-598.
86. Olsen, S., *Brucellosis in the United States: role and significance of wildlife reservoirs.* Vaccine, 2010. **28**: p. 73-76.
87. Cheville, N.F., D.R. McCullough, and L.R. Paulson, *Brucellosis in the greater Yellowstone area*. 1998: National Academies Press.
88. Pappas, G., *The changing Brucella ecology: novel reservoirs, new threats.* International Journal of Antimicrobial Agents, 2010. **36**: p. S8-S11.
89. Mateu-de-Antonio, E. and M. Martin, *In vitro efficacy of several antimicrobial combinations against Brucella canis and Brucella melitensis strains isolated from dogs.* Veterinary Microbiology, 1995. **45**(1): p. 1-10.
90. Blasco, J.M. and B. Molina-Flores, *Control and eradication of Brucella melitensis infection in sheep and goats.* Veterinary Clinics of North America: Food Animal Practice, 2011. **27**(1): p. 95-104.
91. Olsen, S. and M. Palmer, *Advancement of knowledge of Brucella over the past 50 years.* Veterinary Pathology Online, 2014. **1**(14): p. 0300985814540545.
92. Kwa, B., *Environmental change, development and vector-borne disease: Malaysia's experience with filariasis, scrub typhus and dengue.* Environment, Development and Sustainability, 2008. **10**: p. 209-217.

93. Whitlock, G.C., D.M. Estes, and A.G. Torres, *Glanders: off to the races with Burkholderia mallei.* FEMS Microbiology Letters, 2007. **277**(2): p. 115-122.
94. Hammon, W.M., W.C. Reeves, and G. Sather, *California Encephalitis Virus, a Newly Described Agent II. Isolations and Attempts to Identify and Characterize the Agent.* The Journal of Immunology, 1952. **69**(5): p. 493-510.
95. Hammon, W.M. and W. Reeves, *California encepalitis virus - a newly described agent.* California Medicine, 1952. **77**(5): p. 303.
96. Burgdorfer, W., V.F. Newhouse, and L.A. Thomas, *Isolation of California encephalitis virus from the blood of a snowshoe hare (Lepus americanus) in western Montana.* American Journal of Epidemiology, 1961. **73**(3): p. 344-349.
97. Thompson, W.H. and A.S. Evans, *California encepalitis virus studies in Wisconsin. .* American journal of epidemiology, 1965. **81**(2): p. 230-244.
98. Thompson, W.H., B. Kalfayan, and R.O. Anslow, *Isolation of California encephalitis group virus from a fatal human illness.* American Journal of Epidemiology, 1965. **81**(2): p. 245-253.
99. Ritter, D.G. and E.T. Feltz, *On the natural occurrence of California encephalitis virus and other arboviruses in Alaska.* Canadian Journal of Microbiology, 1974. **20**(10): p. 1359-1366.
100. Eldridge, B.F., et al., *The first reported case of California encephalitis in more than 50 years.* Emerging Infectious Diseases, 2001. **7**(3): p. 451-452.
101. Reisen, W., et al., *Persistence of mosquito-borne viruses in Kern County, California, 1983-1988.* The American Journal of Tropical Medicine and Hygiene, 1990. **43**(4): p. 419-437.
102. Stanford, M.M., S.J. Werden, and G. McFadden, *Myxoma virus in the European rabbit: interactions between the virus and its susceptible host.* Veterinary Research, 2007. **38**(2): p. 299-318.
103. Spiesschaert, B., et al., *The current status and future directions of myxoma virus, a master in immune evasion.* Veterinary Research, 2011. **42**: p. e76.
104. Kerr, P.J. and T.M. Donnelly, *Viral infections of rabbits.* Veterinary Clinics of North America: Exotic Animal Practice, 2013. **16**(2): p. 437-468.
105. McGarry, J., et al., *Zoonotic helminths of urban brown rats (Rattus norvegicus) in the UK: Neglected public health considerations?* Zoonoses and Public Health, 2014: p. 1-9.
106. Altekruse, S.F., et al., *Campylobacter jejuni - an emerging foodborne pathogen.* Emerging Infectious Diseases, 1999. **5**(1): p. 28-35.
107. Klein, B.S., et al., *Campylobacter infection associated with raw milk: An outbreak of gastroenteritis due to Campylobacter jejuni and thermotolerant Campylobacter fetus subsp fetus.* JAMA, 1986. **255**(3): p. 361-364.
108. Graham, R.L. and R.S. Baric, *Recombination, reservoirs, and the modular spike: mechanisms of coronavirus cross-species transmission.* Journal of Virology, 2010. **84**(7): p. 3134-3146.
109. Philippa, J., et al., *Antibodies to selected pathogens in free-ranging terrestrial carnivores and marine mammals in Canada.* The Veterinary Record, 2004. **155**(5): p. 135-140.
110. Choquette, L. and E. Kuyt, *Serological indication of canine distemper and of infectious canine hepatitis in wolves (Canis lupus L.) in northern Canada.* Journal of Wildlife Diseases, 1974. **10**(4): p. 321-324.

111. Garcelon, D.K., R.K. Wayne, and B.J. Gonzales, *A serologic survey of the island fox (Urocyon littoralis) on the Channel Islands, California.* Journal of Wildlife Diseases, 1992. **28**(2): p. 223-229.
112. Hedrick, P.W., R.N. Lee, and C. Buchanan, *Canine parvovirus enteritis, canine distemper, and major histocompatibility complex genetic variation in Mexican wolves.* Journal of Wildlife Diseases, 2003. **39**(4): p. 909-913.
113. Gese, E.M., et al., *Prevalence of antibodies against canine parvovirus and canine distemper virus in wild coyotes in southeastern Colorado.* Journal of Wildlife Diseases, 1991. **27**(2): p. 320-323.
114. Cross, J.H. and V. Basaca-Sevilla, *Experimental transmission of Capillaria philippinensis to birds.* Transactions of the Royal Society of Tropical Medicine and Hygiene, 1983. **77**(4): p. 511-514.
115. Bhaibulaya, M. and S. Indra-Ngarm, *Amaurornis phoenicurus and Ardeola bacchus as experimental definitive hosts for Capillaria philippinensis in Thailand.* International Journal for Parasitology, 1979. **9**(4): p. 321-322.
116. Li, H., et al., *Goats are a potential reservoir for the herpesvirus (MCFV-WTD), causing malignant catarrhal fever in deer.* Journal of Zoo and Wildlife Medicine, 2013. **44**(2): p. 484-486.
117. Li, H., et al., *Malignant Catarrhal Fever: Inching Toward Understanding.* Annu. Rev. Anim. Biosci., 2014. **2**(1): p. 209-233.
118. Menghani, S., et al., *Chandipura Virus: an emerging tropical pathogen.* Acta Tropica, 2012. **124**(1): p. 1-14.
119. Thiberville, S.-D., et al., *Chikungunya fever: Epidemiology, clinical syndrome, pathogenesis and therapy.* Antiviral Research, 2013. **99**(3): p. 345-370.
120. Tsetsarkin, K.A., et al., *Chikungunya virus emergence is constrained in Asia by lineage-specific adaptive landscapes.* Proceedings of the National Academy of Sciences, 2011. **108**(19): p. 7872-7877.
121. Pialoux, G., et al., *Chikungunya, an epidemic arbovirosis.* The Lancet Infectious Diseases, 2007. **7**(5): p. 319-327.
122. Powers, A.M. and C.H. Logue, *Changing patterns of chikungunya virus: re-emergence of a zoonotic arbovirus.* Journal of General Virology, 2007. **88**(9): p. 2363-2377.
123. Harkinezhad, T., T. Geens, and D. Vanrompay, *Chlamydophila psittaci infections in birds: a review with emphasis on zoonotic consequences.* Veterinary Microbiology, 2009. **135**(1): p. 68-77.
124. Smith, K.A., et al., *Compendium of measures to control Chlamydophila psittaci (formerly Chlamydia psittaci) infection among humans (psittacosis) and pet birds, 2005.* Journal of the American Veterinary Medical Association, 2005. **226**(4): p. 532-539.
125. Nelson, R., et al., *Confirmation of Choclo virus as the cause of hantavirus cardiopulmonary syndrome and high serum antibody prevalence in Panama.* Journal of Medical Virology, 2010. **82**(9): p. 1586-1593.
126. Francesconi, F. and O. Lupi, *Myiasis.* Clinical Microbiology Reviews, 2012. **25**(1): p. 79-105.
127. Hall, M., et al., *Genetic diversity of populations of Old World screwworm fly, Chrysomya bezziana, causing traumatic myiasis of livestock in the Gulf region and implications for control by sterile insect technique.* Medical and Veterinary Entomology, 2009. **23**(1): p. 51-58.

128. Zhang, Y., et al., *Prevalence of Clonorchis sinensis infection in freshwater fishes in northeastern China.* Veterinary Parasitology, 2014. **204**(3): p. 209-213.
129. Reichard, R., *Area-wide biological control of disease vectors and agents affecting wildlife.* Revue Scientifique et Technique (International Office of Epizootics), 2002. **21**(1): p. 179-185.
130. Burgdorfer, W. and C.M. Eclund, *Studies on the ecology of Colorado tick fever virus in western Montana.* American Journal of Epidemiology, 1959. **69**(2): p. 127-137.
131. Peel, M.M., et al., *Human lymphadenitis due to Corynebacterium pseudotuberculosis: report of ten cases from Australia and review.* Clinical Infectious Diseases, 1997. **24**(2): p. 185-191.
132. Anderson, D.E., D.M. Rings, and J. Kowalski, *Infection with Corynebacterium pseudotuberculosis in five alpacas.* Journal of the American Veterinary Medical Association, 2004. **225**(11): p. 1743-1747.
133. Chantrey, J., et al., *Cowpox: reservoir hosts and geographic range.* Epidemiology and Infection, 1999. **122**(03): p. 455-460.
134. Begon, M., et al., *Transmission dynamics of a zoonotic pathogen within and between wildlife host species.* Proceedings of the Royal Society of London B: Biological Sciences, 1999. **266**(1432): p. 1939-1945.
135. Angelakis, E. and D. Raoult, *Q fever.* Veterinary microbiology, 2010. **140**(3): p. 297-309.
136. Madariaga, M.G., *Q fever wildlife reservoir.* Emerging Infectious Diseases, 2005. **11**(5): p. 776-777.
137. DePuy, W., et al., *Q fever risk across a dynamic, heterogeneous landscape in Laikipia county, Kenya.* EcoHealth, 2014. **11**(3): p. 429-433.
138. Ergönül, Ö., *Crimean-Congo haemorrhagic fever.* The Lancet Infectious Diseases, 2006. **6**(4): p. 203-214.
139. *Crimean-Congo haemorrhagic fever*. 2013 January 2013 [cited 2015; Available from: http://www.who.int/mediacentre/factsheets/fs208/en/.
140. Emmons, C.W., *Prevalence of Cryptococcus neoformans in pigeon habitats.* Public Health Reports, 1960. **75**(4): p. 362-364.
141. Šlapeta, J., *Cryptosporidiosis and Cryptosporidium species in animals and humans: a thirty colour rainbow?* International Journal for Parasitology, 2013. **43**(12): p. 957-970.
142. Peng, M.M., et al., *Genetic polymorphism among Cryptosporidium parvum isolates: evidence of two distinct human transmission cycles.* Emerging Infectious Diseases, 1997. **3**(4): p. 567-573.
143. Webster, J. and D. MacDonald, *Cryptosporidiosis reservoir in whild brown rats (Rattus norvegicus) in the UK.* Epidemiology and Infections, 1995. **115**: p. 207-209.
144. Quy, R.J., et al., *The Norway rat as a reservoir host of Cryptosporidium parvum.* Journal of Wildlife Diseases, 1999. **35**(4): p. 660-670.
145. Peng, M.A., et al., *Genetic polymorphism among Cryptosporidium parvum isolates: Evidence of two distinct human transmission cycles.* Emerging Infectious Diseases, 1997. **3**(4): p. 567-573.
146. Butfiloski, J.W., *Distribution and prevalence of Cytauxzoon felis in bobcats (Lynx rufus), the natural reservoir; and other wild felids in thirteen states.* Veterinary Parasitology, 2010. **175**(3-4): p. 325-330.
147. Hanley, K.A., et al., *Fever versus fever: the role of host and vector susceptibility and interspecific competition in shaping the current and future distributions of the sylvatic*

cycles of dengue virus and yellow fever virus. Infection, Genetics and Evolution, 2013. **19**: p. 292-311.
148. Martinez, D. and P. Prior, *Survival of Dermatophilus congolensis in tropical clay soils submitted to different water potentials.* Veterinary Microbiology, 1991. **29**(2): p. 135-145.
149. Zaria, L., *Dermatophilus congolensis infection (dermatophilosis) in animals and man! An update.* Comparative Immunology, Microbiology and Infectious Diseases, 1993. **16**(3): p. 179-222.
150. Sun, T., A. Turnbull, and P.H. Lieberman, *Giant kidney worm (Dioctophyma renale) infection mimicking retroperitoneal neoplasm.* The American Journal of Surgical Pathology, 1986. **10**(7): p. 508-512.
151. von Bonsdorff, B. and G. Bylund, *The ecology of Diphyllobothrium latum.* Ecology of Disease, 1982. **1**(1): p. 21-26.
152. Morsy, T., S. Michael, and A. El Disi, *Cats as reservoir hosts of human parasites in Amman, Jordan.* Journal of the Egyptian Society of Parasitology, 1980. **10**(1): p. 5-18.
153. Klimpel, S., et al., *Gastrointestinal and ectoparasites from urban stray dogs in Fortaleza (Brazil): high infection risk for humans?* Parasitology Research, 2010. **107**(3): p. 713-719.
154. Magi, M., et al., *Vulpes vulpes: a possible wild reservoir for zoonotic filariae.* Vector-Borne and Zoonotic Diseases, 2008. **8**(2): p. 249-252.
155. Kramer, L.H., et al., *Human subcutaneous dirofilariasis, Russia.* Emerging Infectious Diseases, 2007. **13**(1): p. 150-152.
156. Oktem, I.M.A., et al., *Dobrava-Belgrade virus in Apodemus flavicollis and A. uralensis mice, Turkey.* Emerging Infectious Diseases, 2014. **20**(1): p. 121-125.
157. Schlegel, M., et al., *Dobrava-Belgrade virus spillover infections, Germany.* Emerging Infectious Diseases, 2009. **15**(12): p. 2017-2020.
158. Klempa, B., et al., *Complex evolution and epidemiology of Dobrava-Belgrade hantavirus: definition of genotypes and their characteristics.* Archives of Virology, 2013. **158**(3): p. 521-529.
159. Burt, F., et al., *Investigation of tick-borne viruses as pathogens of humans in South Africa and evidence of Dugbe virus infection in a patient with prolonged thrombocytopenia.* Epidemiology and Infection, 1996. **116**(03): p. 353-361.
160. Rodrigues, R., et al., *Development of a one step real time RT-PCR assay to detect and quantify Dugbe virus.* Journal of Virological Methods, 2011. **176**(1): p. 74-77.
161. Lwande, O.W., et al., *Isolation of tick and mosquito-borne arboviruses from ticks sampled from livestock and wild animal hosts in Ijara District, Kenya.* Vector-Borne and Zoonotic Diseases, 2013. **13**(9): p. 637-642.
162. Calisher, C.H., *Medically important arboviruses of the United States and Canada.* Clinical Microbiology Reviews, 1994. **7**(1): p. 89-116.
163. Scofi, T.W. and S.C. Weover, *Eastern equine encephalomyelitis virus: epidemiology and evolution of mosquito transmission*, in *Advances in Virus Research*. 1990, Academic Press Inc. p. 277-328.
164. Eckert, J. and R. Thompson, *Intraspecific variation of Echinococcus granulosus and related species with emphasis on their infectivity to humans.* Acta Tropica, 1997. **64**(1): p. 19-34.

165. Knapp, J., et al., *Echinococcus vogeli infection in a hunter, French Guiana.* Emerging Infectious Diseases, 2009. **15**(12): p. 2029-2031.
166. Otero-Abad, B. and P.R. Torgerson, *A systematic review of the epidemiology of echinococcosis in domestic and wild animals.* PLoS: Negelcted Tropical Diseases, 2013. **7**(6): p. e2249.
167. Basset, D., et al., *Neotropical echinococcosis in Suriname: Echinococcus oligarthrus in the orbit and Echinococcus vogeli in the abdomen.* The American Journal of Tropical Medicine and Hygiene, 1998. **59**(5): p. 787-790.
168. Chantima, K., J.-Y. Chai, and C. Wongsawad, *Echinostoma revolutum: Freshwater snails as the second intermediate hosts in Chiang Mai, Thailand.* Korean Journal of Parasitology, 2013. **51**(2): p. 183-189.
169. Kanev, I., *Life-cycle, delimitation and redescription of Echinostoma revolutum (Froelich, 1802)(Trematoda: Echinostomatidae).* Systematic Parasitology, 1994. **28**(2): p. 125-144.
170. Allan, B.F., *The effects of forest management practices on human risk of exposure to tick-borne diseases*. 2009, St Louis: Washington University Press.
171. Arens, M.Q., et al., *Detection of Ehrlichia spp. in the blood of wild white-tailed deer in Missouri by PCR assay and serologic analysis.* Journal of Clinical Microbiology, 2003. **41**(3): p. 1263-1265.
172. Ewing, S., et al., *Experimental transmission of Ehrlichia chaffeensis (Rickettsiales: Ehrlichieae) among white-tailed deer by Amblyomma americanum (Acari: Ixodidae).* Journal of Medical Entomology, 1995. **32**(3): p. 368-374.
173. Varela-Stokes, A.S., *Transmission of bacterial agents from lone star ticks to white-tailed deer.* Journal of Medical Entomology, 2007. **44**(3): p. 478-483.
174. Paddock, C.D. and M.J. Yabsley, *Ecological havoc, the rise of white-tailed deer, and the emergence of Amblyomma americanum-associated zoonoses in the United States*, in *Wildlife and Emerging Zoonotic Diseases: the Biology, Circumstances and Consequences of Cross-species Transmission*. 2007, Springer. p. 289-324.
175. Yabsley, M.J., et al., *Ehrlichia ewingii infection in white-tailed deer (Odocoileus virginianus).* Emerging Infectious Diseases, 2002. **8**(7): p. 668-671.
176. Lobanov, V., et al., *Molecular study of free-ranging mule deer and white-tailed deer from British Columbia, Canada, for evidence of Anaplasma spp. and Ehrlichia spp.* Transboundary and Emerging Diseases, 2012. **59**(3): p. 233-243.
177. Peter, T.F., M.J. Burridge, and S.M. Mahan, *Ehrlichia ruminantium infection (heartwater) in wild animals.* Trends in Parasitology, 2002. **18**(5): p. 214-218.
178. Solaymani-Mohammadi, S., et al., *Intestinal protozoa in wild boars (Sus scrofa) in western Iran.* Journal of Wildlife Diseases, 2004. **40**(4): p. 801-803.
179. Martinez, A., et al., *Serosurvey for selected disease agents in white-tailed deer from Mexico.* Journal of Wildlife Diseases, 1999. **35**(4): p. 799-803.
180. Kedmi, M., et al., *No evidence for involvement of sheep in the epidemiology of cattle virulent epizootic hemorrhagic disease virus.* Veterinary Microbiology, 2011. **148**(2): p. 408-412.
181. Ruder, M.G., et al., *Susceptibility of white-tailed deer (Odocoileus virginianus) to experimental infection with epizootic hemorrhagic disease virus serotype 7.* Journal of Wildlife Diseases, 2012. **48**(3): p. 676-685.
182. Vicente, J., et al., *Antibodies to selected viral and bacterial pathogens in European wild boars from southcentral Spain.* Journal of Wildlife Diseases, 2002. **38**(3): p. 649-652.

183. Eriksson, H., et al., *Characterization of Erysipelothrix rhusiopathiae isolates from poultry, pigs, emus, the poultry red mite and other animals.* Veterinary Microbiology, 2009. **137**(1): p. 98-104.
184. Besser, M., Richard E, M. Griffin, Patricia M, and M. Slutsker, MPH, Laurence, *Escherichia coli o157: h7 gastroenteritis and the hemolytic uremic syndrome: an emerging infectious disease.* Annual Review of Medicine, 1999. **50**(1): p. 355-367.
185. Franz, E., et al., *Exploiting the explosion of information associated with whole genome sequencing to tackle Shiga toxin-producing Escherichia coli (STEC) in global food production systems.* International Journal of Food Microbiology, 2014. **187**: p. 57-72.
186. Pal, M., M. Abdurahman, and M. Zewdu, *Growing significance of fascioliasis as an emerging zoonosis.* Life, 2014. **1**(4): p. 10-13.
187. Graczyk, T.K., R.H. Gilman, and B. Fried, *Fasciolopsiasis: is it a controllable food-borne disease?* Parasitology Research, 2001. **87**(1): p. 80-83.
188. Radford, A.D., et al., *Feline calicivirus.* Veterinary Research, 2007. **38**(2): p. 319-335.
189. Benetka, V., et al., *Prevalence of feline coronavirus types I and II in cats with histopathologically verified feline infectious peritonitis.* Veterinary Microbiology, 2004. **99**(1): p. 31-42.
190. Yamaguchi, N., et al., *Parasite prevalence in free-ranging farm cats, Felis silvestris catus.* Epidemiology and Infection, 1996. **116**(02): p. 217-223.
191. Bendinelli, M., et al., *Feline immunodeficiency virus: an interesting model for AIDS studies and an important cat pathogen.* Clinical Microbiology Reviews, 1995. **8**(1): p. 87-112.
192. Lee, I.T., et al., *Prevalence of feline leukemia virus infection and serum antibodies against feline immunodeficiency virus in unowned free-roaming cats.* Journal of the American Veterinary Medical Association, 2002. **220**(5): p. 620-622.
193. Levy, J., et al., *Infectious diseases of dogs and cats on Isabela Island, Galapagos.* Journal of Veterinary Internal Medicine, 2008. **22**(1): p. 60-65.
194. Ward, M.P., S.W. Laffan, and L.D. Highfield, *The potential role of wild and feral animals as reservoirs of foot-and-mouth disease.* Preventive Veterinary Medicine, 2007. **80**(1): p. 9-23.
195. Grubman, M.J. and B. Baxt, *Foot-and-mouth disease.* Clinical Microbiology Reviews, 2004. **17**(2): p. 465-493.
196. Buckley, J., *Observations on Gastrodiscoides hominis and Fasciolopsis buski in Assam.* Journal of Helminthology, 1939. **17**(1): p. 1-12.
197. Mas-Coma, S., M. Bargues, and M. Valero, *Gastrodiscoidiasis, a plant-borne zoonotic disease caused by the intestinal amphistome fluke Gastrodiscoides hominis (Trematoda: Gastrodiscidae).* Revista Iberica de Parasitologia., 2006. **66**: p. 75-81.
198. Cole, R.A., et al., *Gnathostoma spinigerum in live Asian swamp eels (Monopterus spp.) from food markets and wild populations, United States.* Emerging Infectious Diseases, 2014. **20**(4): p. 634-642.
199. Kudo, N., et al., *Experimental infection of laboratory animals and sheep with Gongylonema pulchrum in Japan.* Journal of Veterinary Medical Science, 2003. **65**(8): p. 921-925.
200. Prestwood, A.K., S.R. Pursglove, and F.A. Hayes, *Parasitism among white-tailed deer and domestic sheep on common range.* Journal of Wildlife Diseases, 1976. **12**(3): p. 380-385.

201. Sato, H., *Biology and transmission of the gullet worm (Gongylonema pulchrum Molin, 1857).* Yamaguchi Journal of Veterinary Medicine, 2009. **36**: p. 31-54.
202. Milazzo, M.L., et al., *Transmission of Guanarito and Pirital viruses among wild rodents, Venezuela.* Emerging Infectious Diseases, 2011. **17**(12): p. 2209-2215.
203. Gómez, R.M., et al., *Junin virus. A XXI century update.* Microbes and Infection, 2011. **13**(4): p. 303-311.
204. Davidsen, J., et al., *Can sea trout Salmo trutta compromise successful eradication of Gyrodactylus salaris by hiding from CFT Legumin (rotenone) treatments?* Journal of fish biology, 2013. **82**(4): p. 1411-1418.
205. Robertsen, G., et al., *Arctic charr (Salvelinus alpinus) is a suitable host for Gyrodactylus salaris (Monogenea, Gyrodactylidae) in Norway.* Parasitology, 2007. **134**(2): p. 257-267.
206. Kariwa, H., et al., *A comparative epidemiological study of hantavirus infection in Japan and Far East Russia.* Japanese Journal of Veterinary Research, 2007. **54**(4): p. 145-161.
207. Wang, Z., et al., *Haplosporidium nelsoni and H. costale in the Pacific oyster Crassostrea gigas from China's coasts.* Diseases of Aquatic Organisms, 2010. **89**(3): p. 223-228.
208. Barber, R.D., S.A. Kanaley, and S.E. Ford, *Evidence for regular sporulation by Haplosporidium nelsoni (MSX)(Ascetospora; Haplosporidiidae) in spat of the American oyster, Crassostrea virginica.* The Journal of Protozoology, 1991. **38**(4): p. 305-306.
209. Burreson, E.M. and S.E. Ford, *A review of recent information on the Haplosporidia, with special reference to Haplosporidium nelsoni (MSX disease).* Aquatic Living Resources, 2004. **17**(04): p. 499-517.
210. Ford, S.E. and D. Bushek, *Development of resistance to an introduced marine pathogen by a native host.* Journal of Marine Research, 2012. **70**(2-3): p. 205-223.
211. Ford, S.E., E. Scarpa, and D. Bushek, *Spatial and temporal variability of disease refuges in an estuary: Implications for the development of resistance.* Journal of Marine Research, 2012. **70**(2-3): p. 253-277.
212. Plowright, R.K., et al., *Urban habituation, ecological connectivity and epidemic dampening: the emergence of Hendra virus from flying foxes (Pteropus spp.).* Proceedings of the Royal Society of London B: Biological Sciences, 2011. **278**(1725): p. 3703-3712.
213. Lara, J., M.A. Purdy, and Y.E. Khudyakov, *Genetic host specificity of hepatitis E virus.* Infection, Genetics and Evolution, 2014. **24**: p. 127-139.
214. Johne, R., et al., *Hepeviridae: an expanding family of vertebrate viruses.* Infection, Genetics and Evolution, 2014. **27**: p. 212-229.
215. Meng, X., *Hepatitis E virus: animal reservoirs and zoonotic risk.* Veterinary Microbiology, 2010. **140**(3): p. 256-265.
216. Baneth, G., et al., *Transmission of Hepatozoon canis to dogs by naturally-fed or percutaneously-injected Rhipicephalus sanguineus ticks.* The Journal of Parasitology, 2009. **87**(3): p. 606-611.
217. Baneth, G., M. Samish, and V. Shkap, *Life cycle of Hepatozoon canis (Apicomplexa: Adeleorina: Hepatozoidae) in the tick Rhipicephalus sanguineus and domestic dog (Canis familiaris).* Journal of Parasitology, 2007. **93**(2): p. 283-299.
218. Ramos, R.A.N., et al., *Occurrence of Hepatozoon canis and Cercopithifilaria bainae in an off-host population of Rhipicephalus sanguineus sensu lato ticks.* Ticks and Tick-borne Diseases, 2014. **5**(3): p. 311-314.

219. Rubini, A.S., et al., *Molecular characterization of feline Hepatozoon species from Brazil.* Veterinary Parasitology, 2006. **137**(1): p. 168-171.
220. Gabrielli, S., et al., *The first report of Hepatozoon canis identified in Vulpes vulpes and ticks from Italy.* Vector-borne and Zoonotic Diseases, 2010. **10**(9): p. 855-859.
221. Dežđek, D., et al., *Molecular detection of Theileria annae and Hepatozoon canis in foxes (Vulpes vulpes) in Croatia.* Veterinary Parasitology, 2010. **172**(3): p. 333-336.
222. Baneth, G., et al., *Canine hepatozoonosis: two disease syndromes caused by separate Hepatozoon spp.* TRENDS in Parasitology, 2003. **19**(1): p. 27-31.
223. *B Virus (herpes B, monkey B virus, herpesvirus simiae, and herpesvirus B)*. 2014 July 18, 2014 [cited 2015.
224. Lund, E.E. and A.M. Chute, *The ring-necked pheasant (Phasianus colchicus torquatus) as a host for Heterakis gallinarum and Histomonas meleagridis.* American Midland Naturalist, 1972. **87**(1): p. 1-7.
225. Tattersall, F., F. Nowell, and R. Smith, *A review of the endoparasites of wild house mice Mus domesticus.* Mammal Review, 1994. **24**(2): p. 61-71.
226. Riley, W.A. and W. Shannon, *The rat tapeworm, Hymenolepis diminuta, in man.* The Journal of Parasitology, 1922. **8**(3): p. 109-117.
227. Spackman, E., et al., *Phylogenetic analyses of type A influenza genes in natural reservoir species in North America reveals genetic variation.* Virus Research, 2005. **114**(1): p. 89-100.
228. *Transmission of Influenza Viruses from Animals to People*. Influenza (Flu) 2014 August 19, 2014 [cited 2015.
229. Sonnleitner, S.T., et al., *Investigations on California serogroup orthobunyaviruses in the Tyrols: first description of Tahyna virus in the Alps.* Vector-Borne and Zoonotic Diseases, 2014. **14**(4): p. 272-277.
230. Hubálek, Z., *Mosquito-borne viruses in Europe.* Parasitology Research, 2008. **103**(1): p. 29-43.
231. *Human Jamestown canyon virus infection-Montana, 2009*. Morbidity and Mortality Weekly Report, 2011. **60**, 652-655.
232. Andreadis, T.G., et al., *Isolations of Jamestown Canyon virus (Bunyaviridae: Orthobunyavirus) from field-collected mosquitoes (Diptera: Culicidae) in Connecticut, USA: a ten-year analysis, 1997-2006.* Vector-Borne and Zoonotic Diseases, 2008. **8**(2): p. 175-188.
233. Grimstad, P.R., D.G. Williams, and S.M. Schmitt, *Infection of white-tailed deer (Odocoileus virginianus) in Michigan with Jamestown Canyon virus (California serogroup) and the importance of maternal antibody in viral maintenance.* Journal of Wildlife Diseases, 1987. **23**(1): p. 12-22.
234. Zamparo, J.M., et al., *Serologic evidence of Jamestown Canyon virus infection in white-tailed deer populations from Connecticut.* Journal of Wildlife Diseases, 1997. **33**(3): p. 623-627.
235. Le Flohic, G., et al., *Review of climate, landscape, and viral genetics as drivers of the Japanese encephalitis virus ecology.* PLoS: Neglected Tropical Diseases, 2013. **7**(9): p. e2208.
236. *Japanese Encephalitis: Fact sheet No 386* 2014 [cited 2015.
237. Guterres, A., et al., *Characterization of Juquitiba Virus in Oligoryzomys fornesi from Brazilian Cerrado.* Viruses, 2014. **6**(4): p. 1473-1482.

238. Le Duc, J., et al., *Ecology of California encephalitis viruses on the Del Mar Va Peninsula. II. Demonstration of transovarial transmission.* The American Journal of Tropical Medicine and Hygiene, 1975. **24**(1): p. 124-126.
239. Watts, D.M., et al., *Serologic evidence of Jamestown Canyon and Keystone virus infection in vertebrates of the DelMarVa Peninsula.* The American Journal of Tropical Medicine and Hygiene, 1982. **31**(6): p. 1245-1251.
240. Mackenzie, J., et al., *Arboviruses causing human disease in the Australasian zoogeographic region.* Archives of Virology, 1994. **136**(3-4): p. 447-467.
241. Pattnaik, P., *Kyasanur forest disease: an epidemiological view in India.* Reviews in Medical Virology, 2006. **16**(3): p. 151-165.
242. Holbrook, M.R., *Kyasanur forest disease.* Antiviral research, 2012. **96**(3): p. 353-362.
243. John, J.K., et al., *Kyasanur Forest Disease: a status update.* Advances in Animal and Veterinary Sciences, 2014. **2**(6): p. 329-336.
244. Soldan, S. and F. González-Scarano, *The bunyaviridae.* Handbook of Clinical Neurology, 2013. **123**: p. 449-463.
245. de Moura, M.Q., et al., *First report of Lagochilascaris (Nematoda: Ascarididae) eggs in a public park in Southern Brazil.* Veterinary Parasitology, 2012. **184**(2): p. 359-361.
246. Barrera-Pérez, M., et al., *Lagochilascaris minor Leiper, 1909 (Nematoda: Ascarididae) in Mexico: three clinical cases from the Peninsula of Yucatan.* Revista do Instituto de Medicina Tropical de São Paulo, 2012. **54**(6): p. 315-317.
247. Sakamoto, T. and P.A. Cabrera, *Subcutaneous infection of Lagochilascaris minor in domestic cats from Uruguay.* Veterinary Parasitology, 2002. **108**(2): p. 145-152.
248. Lecompte, E., et al., *Mastomys natalensis and lassa fever, West Africa.* Emerging Infectious Diseases, 2006. **12**(12): p. 1971-1974.
249. *Lassa Fever*. 2015 June 2, 2015 [cited 2015.
250. Mills, J.N., et al., *A longitudinal study of hantavirus infection in three sympatric reservoir species in agroecosystems on the Argentine Pampa.* Vector-Borne and Zoonotic Diseases, 2007. **7**(2): p. 229-240.
251. Gramiccia, M. and L. Gradoni, *The current status of zoonotic leishmaniases and approaches to disease control.* International Journal for Parasitology, 2005. **35**(11): p. 1169-1180.
252. Lemma, W., et al., *A zoonotic focus of cutaneous leishmaniasis in Addis Ababa, Ethiopia.* Parasites and Vectors, 2009. **2**(1): p. 60-60.
253. Saliba, E.K. and O.Y. Oumeish, *Reservoir hosts of cutaneous leishmaniasis.* Clinics in Dermatology, 1999. **17**(3): p. 275-277.
254. Arias, J., et al., *The opossum, Didelphis marsupialis (Marsupialia: Didelphidae), as a reservoir host of Leishmania braziliensis guyanensis in the Amazon Basin of Brazil.* Transactions of the Royal Society of Tropical Medicine and Hygiene, 1981. **75**(4): p. 537-541.
255. Ocampo, C., et al., *Environmental factors associated with American cutaneous leishmaniasis in a new Andean focus in Colombia.* Tropical Medicine and International Health, 2012. **17**(10): p. 1309-1317.
256. Dantas-Torres, F., *The role of dogs as reservoirs of Leishmania parasites, with emphasis on Leishmania (Leishmania) infantum and Leishmania (Viannia) braziliensis.* Veterinary Parasitology, 2007. **149**(3): p. 139-146.

257. Brandão-Filho, S.P., et al., *Wild and synanthropic hosts of Leishmania (Viannia) braziliensis in the endemic cutaneous leishmaniasis locality of Amaraji, Pernambuco State, Brazil.* Transactions of the Royal Society of Tropical Medicine and Hygiene, 2003. **97**(3): p. 291-296.
258. *Leishmaniasis: Fact sheet N°375*. Media Centre 2015 [cited 2015.
259. Acosta, I.D.C., et al., *Survey of Trypanosoma and Leishmania in wild and domestic animals in an Atlantic rainforest fragment and surroundings in the state of Espírito Santo, Brazil.* Journal of Medical Entomology, 2014. **51**(3): p. 686-693.
260. Chajbullinova, A., et al., *The development of Leishmania turanica in sand flies and competition with L. major.* Parasites and Vectors, 2012. **5**: p. e219.
261. Matthias, M.A., et al., *Human leptospirosis caused by a new, antigenically unique Leptospira associated with a Rattus species reservoir in the Peruvian Amazon.* PLoS: Neglected Tropical Diseases, 2008. **2**(4): p. e213.
262. Bharti, A.R., et al., *Leptospirosis: a zoonotic disease of global importance.* The Lancet Infectious Diseases, 2003. **3**(12): p. 757-771.
263. Adler, B. and A. de la Peña Moctezuma, *Leptospira and leptospirosis.* Veterinary Microbiology, 2010. **140**(3): p. 287-296.
264. Griffiths, R. and K. Sinclair. *The fox as a reservoir of Linguatula serrata in Great Britain*. in *Transactions of the Royal Society of Tropical Medicine and Hygiene*. 1953. 26 Portland Place, London, England: Royal Society of Tropical Medicine Manson House
265. Moore, E.R. and G.C. Moore, *The helminth parasites of cottontail rabbits in Alabama, with notes on the arthropod Linguatula serrata.* Journal of Mammalogy, 1947. **28**(3): p. 279-284.
266. Khalafalla, R.E., *A survey study on gastrointestinal parasites of stray cats in northern region of Nile delta, Egypt.* PLoS One, 2011. **6**(7): p. e20283.
267. Laurenson, M.K., et al., *Identifying disease reservoirs in complex systems: mountain hares as reservoirs of ticks and louping-ill virus, pathogens of Red Grouse.* Journal of Animal Ecology, 2003. **72**(1): p. 177-185.
268. Jeffries, C., et al., *Louping ill virus: an endemic tick-borne disease of Great Britain.* Journal of General Virology, 2014. **95**(5): p. 1005-1014.
269. Cope, D.R., G.R. Iason, and I.J. Gordon, *Disease reservoirs in complex systems: a comment on recent work by Laurenson et al.* Journal of Animal Ecology, 2004. **73**(4): p. 807-810.
270. Tuppurainen, E., et al., *Mechanical transmission of lumpy skin disease virus by Rhipicephalus appendiculatus male ticks.* Epidemiology and Infection, 2013. **141**(02): p. 425-430.
271. Lubinga, J., et al., *Evidence of lumpy skin disease virus over-wintering by transstadial persistence in Amblyomma hebraeum and transovarial persistence in Rhipicephalus decoloratus ticks.* Experimental and Applied Acarology, 2014. **62**(1): p. 77-90.
272. Tageldin, M.H., et al., *Lumpy skin disease of cattle: an emerging problem in the Sultanate of Oman.* Tropical Animal Health and Production, 2014. **46**(1): p. 241-246.
273. Forbes, K.M., et al., *Serological survey of rodent-borne viruses in Finnish field voles.* Vector-Borne and Zoonotic Diseases, 2014. **14**(4): p. 278-283.
274. Armstrong, C., J. Wallace, and L. Ross, *Lymphocytic choriomeningitis: gray mice, Mus musculus, a reservoir for the infection.* Public Health Reports, 1940. **55**(27): p. 1222-1229.

275. Kilgore, P.E., et al., *Prospects for the control of Bolivian hemorrhagic fever.* Emerging Infectious Diseases, 1995. **1**(3): p. 97-100.
276. Swanepoel, R., et al., *Studies of reservoir hosts for Marburg virus.* Emerging Infectious Diseases, 2007. **13**(12): p. 1847-1851.
277. Towner, J.S., et al., *Marburg virus infection detected in a common African bat.* PLoS One, 2007. **2**(8): p. e764.
278. Changula, K., et al., *Ebola and Marburg virus diseases in Africa: Increased risk of outbreaks in previously unaffected areas?* Microbiology and immunology, 2014. **58**(9): p. 483-491.
279. Amman, B.R., et al., *Seasonal pulses of Marburg virus circulation in juvenile Rousettus aegyptiacus bats coincide with periods of increased risk of human infection.* PLoS: Pathogens, 2012. **8**(10): p. e1002877.
280. Barr, J., et al., *Isolation of multiple novel paramyxoviruses from pteropid bat urine.* Journal of General Virology, 2015. **96**(1): p. 24-29.
281. Barr, J.A., et al., *Evidence of bat origin for Menangle virus, a zoonotic paramyxovirus first isolated from diseased pigs.* Journal of General Virology, 2012. **93**(12): p. 2590-2594.
282. Philbey, A., et al., *Infection with Menangle virus in flying foxes (Pteropus spp.) in Australia.* Australian Veterinary Journal, 2008. **86**(11): p. 449-454.
283. Ahn, Y.K., *Epidemiological studies on Metagonimus yokogawai infection in Samcheok-gun, Kangwon-do, Korea.* The Korean journal of parasitology, 1984. **22**(2): p. 161-170.
284. Hwang, J.T. and D.W. Choi, *Metacercarial density of Metagonimus yokogawai in Plecoglossus altivelis in Kyungpook province, Korea.* Korean Journal of Parasitology, 1977. **15**(1): p. 30-35.
285. Muto, S., *Studies on human parasites in fresh water fishes (in Lake Biwa) which serve as intermediate hosts, especially on Metagonimus yokogawai in Cyprinus carpio and Carassius auratus.* Journal of Japanese Association of Gastroenterologic Diseases, 1917. **16**(2): p. 961-966.
286. MacLean, J.D., et al., *Common-source outbreak of acute infection due to the North American liver fluke Metorchis conjunctus.* The Lancet, 1996. **347**(8995): p. 154-158.
287. Chai, J.-Y., K.D. Murrell, and A.J. Lymbery, *Fish-borne parasitic zoonoses: status and issues.* International Journal for Parasitology, 2005. **35**(11): p. 1233-1254.
288. Cafarchia, C., et al., *Isolation of Microsporum canis from the hair coat of pet dogs and cats belonging to owners diagnosed with M. canis tinea corporis.* Veterinary Dermatology, 2006. **17**(5): p. 327-331.
289. Pier, A. and K. Moriello, *Parasitic relationship between Microsporum canis and the cat.* Medical Mycology, 1997. **36**: p. 271-275.
290. Smith, J., F. Rush-Munro, and M. McCarthy, *Animals as a reservoir of human ringworm in New Zealand.* Australasian Journal of Dermatology, 1969. **10**(3): p. 169-182.
291. Cabañes, F.J., *Dermatophytes in domestic animals.* Revista Iberoamericana de Micología, 2000. **17**: p. 104-108.
292. English, M.P., *The epidemiology of animal ringworm in man.* British Journal of Dermatology, 1972. **86**(8): p. 78-87.
293. Samara, E.M. and K.A. Abdoun, *Concerns about misinterpretation of recent scientific data implicating dromedary camels in epidemiology of Middle East respiratory syndrome (MERS).* MBio, 2014. **5**(4): p. e01430-14.

294. Azhar, E.I., et al., *Detection of the Middle East respiratory syndrome coronavirus genome in an air sample originating from a camel barn owned by an infected patient.* MBio, 2014. **5**(4): p. e01450-14.
295. Sures, B., K. Grube, and H. Taraschewski, *Experimental studies on the lead accumulation in the cestode Hymenolepis diminuta and its final host, Rattus norvegicus.* Ecotoxicology, 2002. **11**(5): p. 365-368.
296. Mafiana, C., M. Osho, and S. Sam-Wobo, *Gastrointestinal helminth parasites of the black rat (Rattus rattus) in Abeokuta, southwest Nigeria.* Journal of Helminthology, 1997. **71**(3): p. 217-220.
297. Meyer, H., et al., *Outbreaks of disease suspected of being due to human monkeypox virus infection in the Democratic Republic of Congo in 2001.* Journal of Clinical Microbiology, 2002. **40**(8): p. 2919-2921.
298. Di Giulio, D.B. and P.B. Eckburg, *Human monkeypox: an emerging zoonosis.* The Lancet Infectious Diseases, 2004. **4**(1): p. 15-25.
299. Hutson, C.L., et al., *Monkeypox zoonotic associations: insights from laboratory evaluation of animals associated with the multi-state US outbreak.* The American Journal of Tropical Medicine and Hygiene, 2007. **76**(4): p. 757-768.
300. Selvey, L.A., et al., *The changing epidemiology of Murray Valley encephalitis in Australia: the 2011 outbreak and a review of the literature.* PLoS Neglected Tropical Diseases, 2014. **8**(1): p. e2656.
301. Beard, P., et al., *Paratuberculosis infection of nonruminant wildlife in Scotland.* Journal of Clinical Microbiology, 2001. **39**(4): p. 1517-1521.
302. Carta, T., et al., *Wildlife and paratuberculosis: a review.* Research in Veterinary Science, 2013. **94**(2): p. 191-197.
303. Nugent, G., et al., *Assessing the effectiveness of tuberculosis management in brushtail possums (Trichosurus vulpecula), through indirect surveillance of Mycobacterium bovis infection using released sentinel pigs.* Veterinary Medicine International, 2014. **Article ID 361634**: p. 1-13.
304. Chambers, M.A., et al., *Bacillus Calmette-Guérin vaccination reduces the severity and progression of tuberculosis in badgers.* Proceedings of the Royal Society of London B: Biological Sciences, 2011. **278**(1713): p. 1913-1920.
305. Gooding, R. and R. Brook, *Modeling and mitigating winter hay bale damage by elk in a low prevalence bovine tuberculosis endemic zone.* Preventive Veterinary Medicine, 2014. **114**(2): p. 123-131.
306. Worobec, S.M., *Treatment of leprosy/Hansen's disease in the early 21st century.* Dermatologic Therapy, 2009. **22**(6): p. 518-537.
307. Skoric, M., et al., *Tuberculous and tuberculoid lesions in free living small terrestrial mammals and the risk of infection to humans and animals: a review.* Veterinarni Medicina (Praha), 2007. **52**(4): p. 144-161.
308. Cavanagh, R., et al., *Mycobacterium microti infection (vole tuberculosis) in wild rodent populations.* Journal of Clinical Microbiology, 2002. **40**(9): p. 3281-3285.
309. Cavanagh, R.D., et al., *Disease dynamics in cyclic populations of field voles (Microtus agrestis): cowpox virus and vole tuberculosis (Mycobacterium microti).* Proceedings of the Royal Society B: Biological Sciences, 2004. **271**(1541): p. 859-867.
310. Burthe, S., et al., *Tuberculosis (Mycobacterium microti) in wild field vole populations.* Parasitology, 2008. **135**(3): p. 309-317.

311. Corrales, J., et al., *Contagious agalactia in small ruminants.* Small Ruminant Research, 2007. **68**(1): p. 154-166.
312. Kumar, A., et al., *Mycoplasma agalactiae, an etiological agent of contagious agalactia in small ruminants: a review.* Veterinary Medicine International, 2014. **Article ID: 286752**: p. 1-13.
313. Janovsky, M., et al., *Mycoplasma conjunctivae infection is self-maintained in the Swiss domestic sheep population.* Veterinary Microbiology, 2001. **83**(1): p. 11-22.
314. Mavrot, F., et al., *Occurrence, quantification, and genotyping of Mycoplasma conjunctivae in wild Caprinae with and without infectious keratoconjunctivitis.* Journal of Wildlife Diseases, 2012. **48**(3): p. 619-631.
315. Dhondt, A.A., et al., *Can American goldfinches function as reservoirs for Mycoplasma gallisepticum?* Journal of Wildlife Diseases, 2013. **49**(1): p. 49-54.
316. Dhondt, A.A., et al., *Diverse wild bird host range of Mycoplasma gallisepticum in eastern North America.* PLoS One, 2014. **9**(7): p. e103553.
317. Besser, T.E., et al., *Survival of bighorn sheep (Ovis canadensis) commingled with domestic sheep (Ovis aries) in the absence of Mycoplasma ovipneumoniae.* Journal of Wildlife Diseases, 2012. **48**(1): p. 168-172.
318. Jacobson, K.C., et al., *Parasite-associated mortality of juvenile Pacific salmon caused by the trematode Nanophyetus salmincola during early marine residence.* Marine Ecology Progress Series, 2008. **354**: p. 235-244.
319. Sandell, T., et al., *Infections by Renibacterium salmoninarum and Nanophyetus salmincola (Chapin) are associated with reduced growth of juvenile Chinook salmon, Oncorhynchus tshawytscha (Walbaum), in the Northeast Pacific Ocean.* Journal of Fish Diseases, 2015. **38**(4): p. 365-378.
320. Chen, J., et al., *Canine and feline parasitic zoonoses in China.* Parasites and Vectors, 2012. **5**(152): p. 1005-1011.
321. Millán, J. and I. Blasco-Costa, *Molecular evidence of shared hookworm Ancylostoma tubaeforme haplotypes between the critically endangered Iberian lynx and sympatric domestic cats.* Veterinary Parasitology, 2012. **186**(3): p. 518-522.
322. Headley, S.A., et al., *Neorickettsia helminthoeca and salmon poisoning disease: a review.* The Veterinary Journal, 2011. **187**(2): p. 165-173.
323. Pusterla, N., et al., *Detection of Neorickettsia risticii from various freshwater snail species collected from a district irrigation canal in Nevada County, California.* The Veterinary Journal, 2013. **197**(2): p. 489-491.
324. Gibson, K.E., et al., *Neorickettsia risticii is vertically transmitted in the trematode Acanthatrium oregonense and horizontally transmitted to bats.* Environmental Microbiology, 2005. **7**(2): p. 203-212.
325. Goodswen, S.J., P.J. Kennedy, and J.T. Ellis, *A review of the infection, genetics, and evolution of Neospora caninum: from the past to the present.* Infection, Genetics and Evolution, 2013. **13**: p. 133-150.
326. Hjelle, B., et al., *Molecular linkage of hantavirus pulmonary syndrome to the white-footed mouse, Peromyscus leucopus: genetic characterization of the M genome of New York virus.* Journal of Virology, 1995. **69**(12): p. 8137-8141.
327. Rahman, S.A., et al., *Risk factors for Nipah virus infection among pteropid bats, Peninsular Malaysia.* Emerging Infectious Diseases, 2013. **19**(1): p. 51-60.

328. Daszak, P., et al., *Interdisciplinary approaches to understanding disease emergence: the past, present, and future drivers of Nipah virus emergence.* Proceedings of the National Academy of Sciences, 2013. **110**(Supplement 1): p. 3681-3688.
329. Rahman, S.A., et al., *Characterization of Nipah virus from naturally infected Pteropus vampyrus bats, Malaysia.* Emerging Infectious Diseases, 2010. **16**(12): p. 1990-1993.
330. Holbrook, M.R., et al., *An animal model for the tickborne flavivirus—Omsk hemorrhagic fever virus.* Journal of Infectious Diseases, 2005. **191**(1): p. 100-108.
331. LeDuc, J.W., *Epidemiology of hemorrhagic fever viruses.* Review of Infectious Diseases, 1989. **11**(Supplement 4): p. S730-S735.
332. Růžek, D., et al., *Omsk haemorrhagic fever.* The Lancet, 2010. **376**(9758): p. 2104-2113.
333. Onmaz, A., et al., *Vectors and vector-borne diseases of horses.* Veterinary Research Communications, 2013. **37**(1): p. 65-81.
334. Otranto, D., et al., *Vector-borne helminths of dogs and humans in Europe.* Parasites and Vectors, 2013. **6**: p. 16.
335. Uni, S., et al., *Acanthocheilonema delicata n. sp. (Nematoda: Filarioidea) from Japanese badgers (Meles anakuma): Description, molecular identification, and Wolbachia screening.* Parasitology International, 2013. **62**(1): p. 14-23.
336. Eberhard, M.L., et al., *Zoonotic Onchocerca lupi infection in a 22-month-old child in Arizona: first report in the United States and a review of the literature.* The American Journal of Tropical Medicine and Hygiene, 2013. **88**(3): p. 601-605.
337. Ozyildiz, Z., et al., *Parasitic aortitis due to Onchocerca armillata in slaughtered cattle in the southeastern region of Turkey.* Kafkas Üniversitesi Veteriner Fakültesi Dergisi, 2013. **19**(4): p. 589-594.
338. Pozio, E., et al., *Opisthorchis felineus, an emerging infection in Italy and its implication for the European Union.* Acta tropica, 2013. **126**(1): p. 54-62.
339. Armignacco, O., et al., *Human illnesses caused by Opisthorchis felineus flukes, Italy.* Emerging Infectious Diseases, 2008. **14**(12): p. 1902-1905.
340. Sithithaworn, P. and M. Haswell-Elkins, *Epidemiology of Opisthorchis viverrini.* Acta tropica, 2003. **88**(3): p. 187-194.
341. Chareonviriyaphap, T., et al., *Dual exposure of Rickettsia typhi and Orientia tsutsugamushi in the field-collected Rattus rodents from Thailand.* Journal of Vector Ecology, 2014. **39**(1): p. 182-189.
342. Kelly, D.J., et al., *Scrub typhus: the geographic distribution of phenotypic and genotypic variants of Orientia tsutsugamushi.* Clinical Infectious Diseases, 2009. **48**(Supplement 3): p. S203-S230.
343. Kim, B.N., L.F. Gordillo, and Y. Kim, *A model for the transmission dynamics of Orientia tsutsugamushi among its natural reservoirs.* Journal of Theoretical Biology, 2010. **266**(1): p. 154-161.
344. Coleman, R.E., et al., *Occurrence of Orientia tsutsugamushi in small mammals from Thailand.* The American Journal of Tropical Medicine and Hygiene, 2003. **69**(5): p. 519-524.
345. Sood, R., et al., *Detection and molecular characterization of naturally transmitted sheep associated malignant catarrhal fever in cattle in India.* Tropical Animal Health and Production, 2014. **46**(6): p. 1037-1043.
346. Russell, G.C., J.P. Stewart, and D.M. Haig, *Malignant catarrhal fever: a review.* The Veterinary Journal, 2009. **179**(3): p. 324-335.

347. Cabrera, B., *Paragonimiasis in the Philippines: current status.* Arzneimittel-Forschung, 1983. **34**(9B): p. 1188-1192.
348. Sohn, W.-M. and J.-Y. Chai, *Infection status with helminthes in feral cats purchased from a market in Busan, Republic of Korea.* The Korean Journal of Parasitology, 2005. **43**(3): p. 93-100.
349. Aktories, K., J.H. Orth, and B. Adler, *Pasteurella multocida: Molecular Biology, Toxins and Infection*. Vol. 361. 2012: Springer Science & Business Media.
350. Banyard, A.C., et al., *Global distribution of peste des petits ruminants virus and prospects for improved diagnosis and control.* Journal of General Virology, 2010. **91**(12): p. 2885-2897.
351. Anderson, E., *Morbillivirus infections in wildlife (in relation to their population biology and disease control in domestic animals).* Veterinary Microbiology, 1995. **44**(2): p. 319-332.
352. Couacy-Hymann, E., et al., *Surveillance of wildlife as a tool for monitoring rinderpest and peste des petits ruminants in West Africa.* Revue Scientifique et Technique-Office International des Epizooties, 2005. **24**(3): p. 869-877.
353. Imai, N., et al., *Transmission and control of Plasmodium knowlesi: a mathematical modelling study.* PLoS Neglected Tropical Diseases, 2014. **8**(7): p. e2978.
354. Lee, K.-S., et al., *Plasmodium knowlesi: reservoir hosts and tracking the emergence in humans and macaques.* PLoS Pathogens, 2011. **7**(4): p. e1002015.
355. Singh, B., et al., *A large focus of naturally acquired Plasmodium knowlesi infections in human beings.* The Lancet, 2004. **363**(9414): p. 1017-1024.
356. Moyes, C.L., et al., *Defining the geographical range of the Plasmodium knowlesi reservoir.* PLoS Neglected Tropical Diseases, 2014. **8**(3): p. e2780.
357. Ebel, G.D., *Update on Powassan virus: emergence of a North American tick-borne flavivirus.* Annual Review of Entomology, 2010. **55**: p. 95-110.
358. Essbauer, S., M. Pfeffer, and H. Meyer, *Zoonotic poxviruses.* Veterinary Microbiology, 2010. **140**(3): p. 229-236.
359. Tersago, K., et al., *Hantavirus outbreak in Western Europe: reservoir host infection dynamics related to human disease patterns.* Epidemiology and Infection, 2011. **139**(03): p. 381-390.
360. Klingström, J., et al., *Rodent host specificity of European hantaviruses: evidence of Puumala virus interspecific spillover.* Journal of Medical Virology, 2002. **68**(4): p. 581-588.
361. Forbes, K.M., et al., *Experimental investigation of a hantavirus host-switch between arvicoline rodents Lemmus lemmus and Myodes glareolus.* Journal of Vector Ecology, 2013. **38**(2): p. 408-410.
362. Kallio, E.R., et al., *Prolonged survival of Puumala hantavirus outside the host: evidence for indirect transmission via the environment.* Journal of General Virology, 2006. **87**(8): p. 2127-2134.
363. Mayes, B.C., et al., *Epidemiology of rabies in bats in Texas (2001–2010).* Journal of the American Veterinary Medical Association, 2013. **243**(8): p. 1129-1137.
364. Davidson, W.R., et al., *Diseases and parasites of red foxes, gray foxes, and coyotes from commercial sources selling to fox-chasing enclosures.* Journal of Wildlife Diseases, 1992. **28**(4): p. 581-589.

365. Gordon, E., et al., *Temporal dynamics of rabies in a wildlife host and the risk of cross-species transmission.* Epidemiology and Infection, 2004. **132**(3): p. 515-524.
366. Lotze, J.-H. and S. Anderson, *Procyon lotor.* Mammalian Species, 1979. **119**: p. 1-8.
367. Kappus, K.D., et al., *The raccoon an emerging rabies host.* Journal of Wildlife Diseases, 1970. **6**(4): p. 507-509.
368. Wandeler, A., et al., *Dog ecology and dog rabies control.* Review of Infectious Diseases, 1988. **10**(Supplement 4): p. S684-S688.
369. Palmer, G.H. and A.F. Azad, *Intracellular Pathogens II: Rickettsiales*. Vol. 2. 2012: American Society for Microbiology Press.
370. Levin, M.L., L.F. Killmaster, and G.E. Zemtsova, *Domestic dogs (Canis familiaris) as reservoir hosts for Rickettsia conorii.* Vector-Borne and Zoonotic Diseases, 2012. **12**(1): p. 28-33.
371. Higgins, J.A., et al., *Rickettsia felis: a new species of pathogenic rickettsia isolated from cat fleas.* Journal of Clinical Microbiology, 1996. **34**(3): p. 671-674.
372. Stenos, J., et al., *Aponomma hydrosauri, the reptile-associated tick reservoir of Rickettsia honei on Flinders Island, Australia.* The American Journal of Tropical Medicine and Hygiene, 2003. **69**(3): p. 314-317.
373. Sonenshine, D.E., et al., *Epizootiology of epidemic typhus (Rickettsia prowazekii) in flying squirrels.* The American Journal of Tropical Medicine and Hygiene, 1978. **27**(2): p. 339-349.
374. Azad, A.F. and C.B. Beard, *Rickettsial pathogens and their arthropod vectors.* Emerging Infectious Diseases, 1998. **4**(2): p. 179-186.
375. Rydkina, E., et al., *New Rickettsiae in ticks collected in territories of the former soviet union.* Emerging Infectious Diseases, 1999. **5**(6): p. 811-814.
376. Kock, R., et al., *Re-infection of wildlife populations with rinderpest virus on the periphery of the Somali ecosystem in East Africa.* Preventive Veterinary Medicine, 2006. **75**(1): p. 63-80.
377. Roeder, P.L., *Rinderpest: The end of cattle plague.* Preventive Veterinary Medicine, 2011. **102**(2): p. 98-106.
378. Plowright, W. *The effects of rinderpest and rinderpest control on wildlife in Africa*. in *Symposia of the Zoological Society of London*. 1982.
379. Rossiter, P. and H. Wamwayi, *Surveillance and monitoring programmes in the control of rinderpest: a review.* Tropical Animal Health and Production, 1989. **21**(2): p. 89-99.
380. Macnish, M., et al., *Detection of the rodent tapeworm Rodentolepis (Hymenolepis) microstoma in humans. A new zoonosis?* International Journal for Parasitology, 2003. **33**(10): p. 1079-1085.
381. Macnish, M., et al., *Failure to infect laboratory rodent hosts with human isolates of Rodentolepis (Hymenolepis) nana.* Journal of Helminthology, 2002. **76**(1): p. 37-43.
382. Potter, A., et al., *The seroprevalence and factors associated with Ross River virus infection in western grey kangaroos (Macropus fuliginosus) in Western Australia.* Vector-Borne and Zoonotic Diseases, 2014. **14**(10): p. 740-745.
383. Russell, R.C., *Ross River virus: ecology and distribution.* Annual Review of Entomology, 2002. **47**(1): p. 1-31.
384. Harley, D., A. Sleigh, and S. Ritchie, *Ross River virus transmission, infection, and disease: a cross-disciplinary review.* Clinical Microbiology Reviews, 2001. **14**(4): p. 909-932.

385. Olsson, G.E., H. Leirs, and H. Henttonen, *Hantaviruses and their hosts in Europe: reservoirs here and there, but not everywhere?* Vector-Borne and Zoonotic Diseases, 2010. **10**(6): p. 549-561.
386. Day, J.F., *Predicting St. Louis encephalitis virus epidemics: lessons from recent, and not so recent, outbreaks.* Annual Review of Entomology, 2001. **46**(1): p. 111-138.
387. Bowen, G.S., et al., *Geographic variation among St. Louis encephalitis virus strains in the viremic responses of avian hosts.* The American Journal of Tropical Medicine and Hygiene, 1980. **29**(6): p. 1411-1419.
388. Navarro-Gonzalez, N., et al., *Effect of cattle on Salmonella carriage, diversity and antimicrobial resistance in free-ranging wild boar (Sus scrofa) in northeastern Spain.* PLoS One, 2012. **7**(12): p. e51614.
389. Jackson, B.R., et al., *Outbreak-associated Salmonella enterica serotypes and food commodities, United States, 1998–2008.* Emerging Infectious Diseases, 2013. **19**(8): p. 1239-1244.
390. Ebani, V.V. and F. Fratini, *Bacterial zoonoses among domestic reptiles.* Annali Della Facoltà di Medicina Veterinaria, 2005. **58**: p. 85-91.
391. Depaquit, J., et al., *Arthropod-borne viruses transmitted by Phlebotomine sandflies in Europe: a review.* Eurosurveillance, 2010. **15**(10): p. e19507.
392. Gakuya, F., et al., *The curse of the prey: Sarcoptes mite molecular analysis reveals potential prey-to-predator parasitic infestation in wild animals from Masai Mara, Kenya.* Parasites and Vectors, 2011. **4**: p. e193.
393. Gakuya, F., et al., *Knowledge of mange among Masai pastoralists in Kenya.* 7, 2012. **8**(e44342).
394. Alasaad, S., et al., *Temporal stability in the genetic structure of Sarcoptes scabiei under the host-taxon law: empirical evidences from wildlife-derived Sarcoptes mite in Asturias, Spain.* Parasites and Vectors, 2011. **4**: p. e151.
395. Oleaga, A., et al., *Sarcoptic mange in red deer from Spain: Improved surveillance or disease emergence?* Veterinary Parasitology, 2008. **154**(1): p. 103-113.
396. León-Vizcaíno, L., et al., *Sarcoptic mange in Spanish ibex from Spain.* Journal of Wildlife Diseases, 1999. **35**(4): p. 647-659.
397. Lavín, S., et al., *Experimental infection of chamois (Rupicapra pyrenaica parva) with Sarcoptes scabiei derived from naturally infected goats.* Journal of Veterinary Medicine, Series B, 2000. **47**(9): p. 693-699.
398. Arlian, L., et al., *Survival and infestivity of Sarcoptes scabiei var. canis and var. hominis.* Journal of the American Academy of Dermatology, 1984. **11**(2): p. 210-215.
399. Walton, S.F., et al., *Genetically distinct dog-derived and human-derived Sarcoptes scabiei in scabies-endemic communities in northern Australia.* The American Journal of Tropical Medicine and Hygiene, 1999. **61**(4): p. 542-547.
400. Gryseels, B., et al., *Human schistosomiasis.* The Lancet, 2006. **368**(9541): p. 1106-1118.
401. Wang, L.-D., et al., *A strategy to control transmission of Schistosoma japonicum in China.* New England Journal of Medicine, 2009. **360**(2): p. 121-128.
402. Strandgaard, H., et al., *The pig as a host for Schistosoma mekongi in Laos.* Journal of Parasitology, 2001. **87**(3): p. 708-709.
403. Lin, X.-D., et al., *Migration of Norway rats resulted in the worldwide distribution of Seoul hantavirus today.* Journal of Virology, 2012. **86**(2): p. 972-981.

404. Dupinay, T., et al., *Detection and genetic characterization of Seoul Virus from commensal brown rats in France.* Virology Journal, 2014. **11**(1): p. e32.
405. Li, W., et al., *Bats are natural reservoirs of SARS-like coronaviruses.* Science, 2005. **310**(5748): p. 676-679.
406. Yan, X.-M., et al., *An outbreak of sheep pox associated with goat poxvirus in Gansu province of China.* Veterinary Microbiology, 2012. **156**(3): p. 425-428.
407. Núñez, J.J., et al., *Hantavirus infections among overnight visitors to Yosemite National Park, California, USA, 2012.* Emerging Infectious Diseases, 2014. **20**(3): p. 386-393.
408. Wiwanitkit, V., *A review of human sparganosis in Thailand.* International Journal of Infectious Diseases, 2005. **9**(6): p. 312-316.
409. Kosoy, M., et al., *Aboriginal and invasive rats of genus Rattus as hosts of infectious agents.* Vector-Borne and Zoonotic Diseases, 2015. **15**(1): p. 3-12.
410. Bacon, M., R.F. Bacon, and C.H. Drake, *Bacterial infections in wild rodents of eastern and central Washington.* Journal of Infectious Diseases, 1958. **102**(1): p. 5-13.
411. Rushton, S., et al., *Disease threats posed by alien species: the role of a poxvirus in the decline of the native red squirrel in Britain.* Epidemiology and Infection, 2006. **134**(3): p. 521-533.
412. Haller, S.L., et al., *Poxviruses and the evolution of host range and virulence.* Infection Genetics and Evolution, 2014. **21**: p. 15-40.
413. Elliott, S.P., *Rat bite fever and Streptobacillus moniliformis.* Clinical Microbiology Reviews, 2007. **20**(1): p. 13-22.
414. Hamburger, M. and H.C. Knowles, *Streptobacillus moniliformis infection complicated by acute bacterial endocarditis: Report of a case in a physician following bite of laboratory rat.* AMA Archives of Internal Medicine, 1953. **92**(2): p. 216-220.
415. Priestnall, S. and K. Erles, *Streptococcus zooepidemicus: an emerging canine pathogen.* The Veterinary Journal, 2011. **188**(2): p. 142-148.
416. Pelkonen, S., et al., *Transmission of Streptococcus equi subspecies zooepidemicus infection from horses to humans.* Emerging Infectious Diseases, 2013. **19**(7): p. 1041-1048.
417. Hughes, J.M., et al., *Streptococcus suis: an emerging human pathogen.* Clinical Infectious Diseases, 2009. **48**(5): p. 617-625.
418. Müller, T., et al., *Pseudorabies virus in wild swine: a global perspective.* Archives of Virology, 2011. **156**(10): p. 1691-1705.
419. Li, W., et al., *Tahyna virus infection, a neglected arboviral disease in the Qinghai-Tibet plateau of China.* Vector-Borne and Zoonotic Diseases, 2014. **14**(5): p. 353-357.
420. Wood, J., et al., *Merogonic stages of Theileria cervi in mule deer (Odocoileus hemionus).* Journal of Veterinary Diagnostic Investigation, 2013. **25**(5): p. 662-665.
421. Gachohi, J., et al., *Epidemiology of East Coast fever (Theileria parva infection) in Kenya: past, present and the future.* Parasites and Vectors, 2012. **5**: p. e194.
422. Chaisi, M.E., N.E. Collins, and M.C. Oosthuizen, *Phylogeny of Theileria buffeli genotypes identified in the South African buffalo (Syncerus caffer) population.* Veterinary Parasitology, 2014. **204**(3): p. 87-95.
423. Bishop, R., et al., *Theileria: intracellular protozoan parasites of wild and domestic ruminants transmitted by ixodid ticks.* Parasitology, 2004. **129**(1): p. 271-283.
424. Stagg, D., et al., *Infection of mammalian cells with Theileria species.* Parasitology, 1983. **86**(2): p. 243-254.

425. Otranto, D., et al., *Current status and epidemiological observation of Thelazia callipaeda (Spirurida, Thelaziidae) in dogs, cats and foxes in Italy: a "coincidence" or a parasitic disease of the Old Continent?* Veterinary Parasitology, 2003. **116**(4): p. 315-325.
426. Otranto, D., et al., *Biology of Thelazia callipaeda (Spirurida, Thelaziidae) eyeworms in naturally infected definitive hosts.* Parasitology, 2004. **129**(05): p. 627-633.
427. Slovák, M., et al., *Survival dynamics of tick-borne encephalitis virus in Ixodes ricinus ticks.* Ticks and Tick-Borne Diseases, 2014. **5**(6): p. 962-969.
428. Macpherson, C.N., *The epidemiology and public health importance of toxocariasis: a zoonosis of global importance.* International Journal for Parasitology, 2013. **43**(12): p. 999-1008.
429. Tenter, A.M., A.R. Heckeroth, and L.M. Weiss, *Toxoplasma gondii: from animals to humans.* International Journal for Parasitology, 2000. **30**(12): p. 1217-1258.
430. Pozio, E., et al., *Hosts and habitats of Trichinella spiralis and Trichinella britovi in Europe.* International Journal for Parasitology, 2009. **39**(1): p. 71-79.
431. Pozio, E., *Searching for Trichinella: not all pigs are created equal.* Trends in Parasitology, 2014. **30**(1): p. 4-11.
432. Takatori, K. and S. Ichijo, *Human dermatophytosis caused by Trichophyton equinum.* Mycopathologia, 1985. **90**(1): p. 15-19.
433. Morris, P. and M.P. English, *Trichophyton mentagrophytes var. erinacei in British hedgehogs.* Sabouraudia, 1969. **7**(2): p. 122-128.
434. English, M.P. and P. Morris, *Trichophyton mentagrophytes var. erinacei in hedgehog nests.* Sabouraudia: Journal of Medical and Veterinary Mycology, 1969. **7**(2): p. 118-121.
435. Papini, R., et al., *High infection rate of Trichophyton verrucosum in calves from Central Italy.* Zoonoses and Public Health, 2009. **56**(2): p. 59-64.
436. Romano, C., et al., *Case reports. Six cases of infection due to Trichophyton verrucosum.* Mycoses, 2001. **44**(7-8): p. 334-337.
437. Rotureau, B. and J. Van Den Abbeele, *Through the dark continent: African trypanosome development in the tsetse fly.* Frontiers in Cellular and Infection Microbiology, 2013. **3**: p. e53.
438. Van den Bossche, P., et al., *A changing environment and the epidemiology of tsetse-transmitted livestock trypanosomiasis.* Trends in Parasitology, 2010. **26**(5): p. 236-243.
439. Van den Bossche, P., et al., *Virulence in Trypanosoma congolense Savannah subgroup. A comparison between strains and transmission cycles.* Parasite Immunology, 2011. **33**(8): p. 456-460.
440. Murray, M., W. Morrison, and D. Whitelaw, *Host susceptibility to African trypanosomiasis: trypanotolerance.* Advances in Parasitology, 1982. **21**: p. 1-68.
441. Anderson, N.E., et al., *Characterisation of the wildlife reservoir community for human and animal trypanosomiasis in the Luangwa Valley, Zambia.* PLoS Neglected Tropical Diseases, 2011. **5**(6): p. e1211.
442. Moloo, S., et al., *A comparison of African buffalo, N'Dama and Boran cattle as reservoirs of Trypanosoma congolense for different Glossina species.* Medical and Veterinary Entomology, 1992. **6**(3): p. 225-230.
443. Hamill, L.C., et al., *Domestic pigs as potential reservoirs of human and animal trypanosomiasis in Northern Tanzania.* Parasites and Vectors, 2013. **6**(1): p. e322.
444. Bern, C., et al., *Trypanosoma cruzi and Chagas' disease in the United States.* Clinical Microbiology Reviews, 2011. **24**(4): p. 655-681.

445. Carabarin-Lima, A., et al., *Chagas disease (American trypanosomiasis) in Mexico: an update.* Acta Tropica, 2013. **127**(2): p. 126-135.
446. Dräger, N. and D. Mehlitz, *Investigations on the prevalence of trypanosome carriers and the antibody response in wildlife in Northern Botswana.* Tropenmedizin und Parasitologie, 1978. **29**(2): p. 223-233.
447. Gardiner, P., *Recent studies of the biology of Trypanosoma vivax.* Advances in Parasitology, 1989. **28**: p. 229-317.
448. Jones, T.W. and A.M. Dávila, *Trypanosoma vivax–out of Africa.* TRENDS in Parasitology, 2001. **17**(2): p. 99-101.
449. Dávila, A., et al., *Using PCR for unraveling the cryptic epizootiology of livestock trypanosomosis in the Pantanal, Brazil.* Veterinary Parasitology, 2003. **117**(1): p. 1-13.
450. Heukelbach, J., et al., *The animal reservoir of Tunga penetrans in severely affected communities of north-east Brazil.* Medical and Veterinary Entomology, 2004. **18**(4): p. 329-335.
451. Weaver, S.C., et al., *Venezuelan equine encephalitis.* Annual Reviews in Entomology, 2004. **49**(1): p. 141-174.
452. McCluskey, B.J., et al., *Vesicular stomatitis outbreak in the southwestern United States, 2012.* Journal of Veterinary Diagnostic Investigation, 2013. **20**(10): p. 1-20.
453. Perez, A.M., et al., *Spatial and phylogenetic analysis of vesicular stomatitis virus over-wintering in the United States.* Preventive Veterinary Medicine, 2010. **93**(4): p. 258-264.
454. Velazquez-Salinas, L., et al., *Phylogeographic characteristics of vesicular stomatitis New Jersey viruses circulating in Mexico from 2005 to 2011 and their relationship to epidemics in the United States.* Virology, 2014. **449**: p. 17-24.
455. Mead, D.G., F.B. Ramberg, and C. MARÉ, *Laboratory vector competence of black flies (Diptera: Simuliidae) for the Indiana serotype of vesicular stomatitis virus.* Annals of the New York Academy of Sciences, 2000. **916**(1): p. 437-443.
456. Blackburn, N. and R. Swanepoel, *An investigation of flavivirus infections of cattle in Zimbabwe Rhodesia with particular reference to Wesselsbron virus.* Journal of Hygiene, 1980. **85**(1): p. 1-33.
457. Neitz, W., *The epidemiological pattern of viral, protophytal and protozoal zoonoses in relation to game preservation in South Africa.* Journal of South African Veterinary Association, 1967. **38**: p. 129-141.
458. Kilpatrick, A.M., et al., *Host heterogeneity dominates West Nile virus transmission.* Proceedings of the Royal Society of London B: Biological Sciences, 2006. **273**(1599): p. 2327-2333.
459. Kilpatrick, A.M., et al., *West Nile virus epidemics in North America are driven by shifts in mosquito feeding behavior.* PLoS Biology, 2006. **4**(4): p. e606.
460. Williams, J.E., et al., *Wild birds as eastern (EEE) and western (WEE) equine encephalitis sentinels.* Journal of Wildlife Diseases, 1971. **7**(3): p. 188-194.
461. Bottone, E.J., *Yersinia enterocolitica: overview and epidemiologic correlates.* Microbes and Infection, 1999. **1**(4): p. 323-333.
462. Valentin-Weigand, P., J. Heesemann, and P. Dersch, *Unique virulence properties of Yersinia enterocolitica O: 3–An emerging zoonotic pathogen using pigs as preferred reservoir host.* International Journal of Medical Microbiology, 2014. **304**(7): p. 824-834.
463. Gage, K.L. and M.Y. Kosoy, *Natural history of plague: perspectives from more than a century of research.* Annual Review of Entomology, 2005. **50**: p. 505-528.

464. Zipser Adjemian, J., et al., *Evidence of multiple zoonotic agents in a wild rodent community in the eastern Sierra Nevada.* Journal of Wildlife Diseases, 2008. **44**(3): p. 737-742.
465. Foley, J.E., et al., *Modeling plague persistence in host-vector communities in California.* Journal of Wildlife Diseases, 2007. **43**(3): p. 408-424.
466. Tollenaere, C., et al., *Susceptibility to Yersinia pestis experimental infection in wild Rattus rattus, reservoir of plague in Madagascar.* EcoHealth, 2010. **7**(2): p. 242-247.
467. Leroy, E.M., et al., *Fruit bats as reservoirs of Ebola virus.* Nature, 2005. **438**(7068): p. 575-576.
468. Pourrut, X., et al., *Spatial and temporal patterns of Zaire ebolavirus antibody prevalence in the possible reservoir bat species.* Journal of Infectious Diseases, 2007. **196**(Supplement 2): p. S176-S183.
469. Groseth, A., H. Feldmann, and J.E. Strong, *The ecology of Ebola virus.* Trends in Microbiology, 2007. **15**(9): p. 408-416.
470. Hayman, D.T., et al., *Ebola virus antibodies in fruit bats, Ghana, West Africa.* Emerging Infectious Diseases, 2012. **18**(7): p. 1207-1209.
471. Gatherer, D., *The 2014 Ebola virus disease outbreak in West Africa.* Journal of General Virology, 2014. **95**(8): p. 1619-1624.
472. Weingartl, H.M., et al., *Transmission of Ebola virus from pigs to non-human primates.* Scientific Reports, 2012. **2**: p. e811.
