# Supplementary material for: Are disease reservoirs special? Taxonomic and life history characteristics
Source: PLoS One. 2017 Jul 13;12(7):e0180716. doi: 10.1371/journal.pone.0180716 (PMC5509157; doi:10.1371/journal.pone.0180716)
Supplement: S2 Text — (PDF) [file pone.0180716.s004.pdf]

S2 Text. Criteria used to assign confidence levels to reservoir identities.

None: Reservoir is implicated, but no diagnostic evidence was available.

Low: Only evidence of natural infection (antibodies, PCR, culture, microscopy) in populations connected to target.

Medium: Direct observation of natural infection in host (visual, PCR, culture or microscopy), plus one additional piece of evidence for **High**.

High: Medium criteria plus empirical evidence that logically supports both that

A) this population or community is the source of most infections of target hosts, and
B) the parasite would be driven extinct with the loss of this population or community.

The evidence for this could come from a wide range of studies, including:

- Experimental infection (i.e. confirmation of susceptibility)
- Confirmed infectiousness in experimental or natural setting
- Changes in reservoir abundance precede and are mimicked by changes in disease incidence (e.g. risk factor analysis)
- Direct measurement of (A), e.g. blood meal analysis of collected vectors, other epidemiological studies of outbreaks
- Elimination of all other possible reservoirs through above studies
