# Supplementary figures and images for: Are disease reservoirs special? Taxonomic and life history characteristics
Source: PLoS One. 2017 Jul 13;12(7):e0180716. doi: 10.1371/journal.pone.0180716 (PMC5509157; doi:10.1371/journal.pone.0180716)

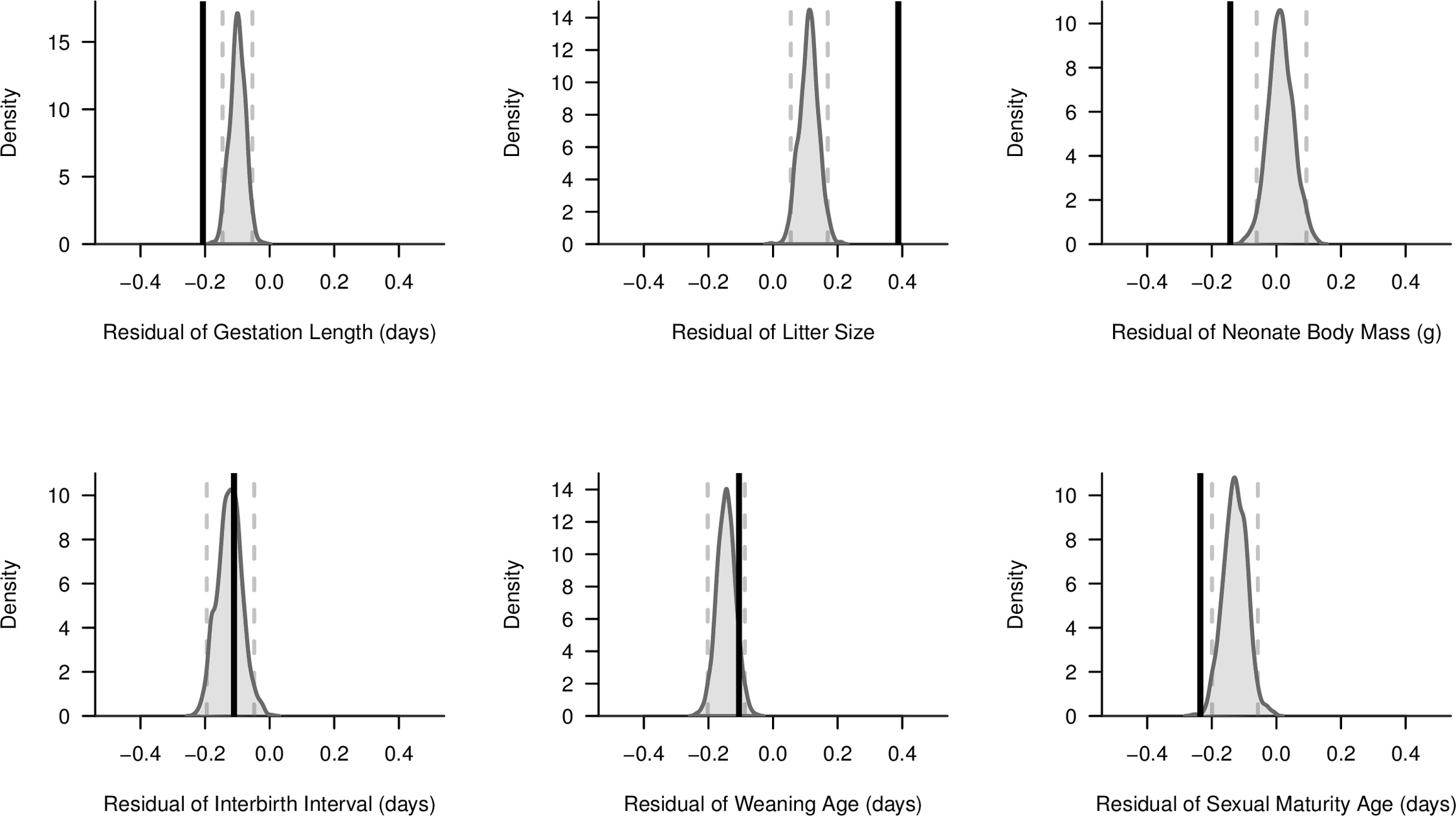

Supplement: S1 Fig — We conducted imputation tests using mass-corrected residual trait values that were generated by regressing six key life history traits on adult body mass (g). To generate an expected distribution of mean residual trait values, we randomly generated 1,000 sets of mammalian species that had the same taxonomic representation (at the order level) as the unique mammalian reservoir species we identified that hosted pathogens with human targets. By calculating the mean residual trait values for these random sets of species, we were able to generate the mean mass-corrected residual trait distributions displayed in grey. The 2.5th and 97.5th percentiles of each distribution are represented with dashed vertical grey lines. For comparison, the observed mean mass-corrected residual trait value of the identified mammalian reservoir species that host human pathogens is shown as a vertical black line. (TIF) [file pone.0180716.s005.tif]
