# Supplementary material for: Are disease reservoirs special? Taxonomic and life history characteristics
Source: PLoS One. 2017 Jul 13;12(7):e0180716. doi: 10.1371/journal.pone.0180716 (PMC5509157; doi:10.1371/journal.pone.0180716)
Supplement: S1 Table — Data show the percentage of disease systems found on each continent. In addition to the pathogen being endemic on a continent, the reservoir and target were also required to occur. Many systems (n = 99) were classified as worldwide and are thus present on each of the listed continents. The number of systems is given in parentheses. *The epidemic potential zone subsets represent the transmission potential of the pathogen in the target host population(s) following spillover. The zones are defined as follows: dead-end for a basic reproductive number (R0) nearly equal to zero, stuttering chains for an R0 greater than zero but less than one, and epidemic potential for an R0 greater than one. †High priority zoonotic pathogen subsets were determined by estimating the pathogen’s representation in the scientific literature using the H-index. Each subset was created to include pathogens that are among the 25% (Top 25%) and 10% (Top 10%) most significant known human pathogens. (PDF) [file pone.0180716.s006.pdf]

**S1 Table. Geographical distribution of disease systems.**

| Region | All Systems (n = 330) | Epidemic Potential Zone* Dead-End (220) | Stuttering Chains (44) | Epidemic Potential (66) | Human Target (261) | High Priority Zoonotic Pathogens† Top 25% (109) | Top 10% (45) |
|---|---|---|---|---|---|---|---|
| North America | 54.24% (179) | 58.64% (129) | 54.55% (24) | 39.39% (26) | 54.79% (143) | 58.72% (64) | 57.78% (26) |
| South America | 42.73% (141) | 45.45% (100) | 45.45% (20) | 31.82% (21 | 44.44% (116) | 39.45% (43) | 33.33% (15) |
| Africa | 46.06% (152) | 42.73% (94) | 59.09% (26) | 48.48% (32) | 44.83% (117) | 47.71% (52) | 48.89% (22) |
| Oceania | 33.33% (110) | 35.91% (79) | 36.36% (16) | 22.73% (15) | 34.87% (91) | 37.61% (41) | 28.89% (13) |
| Europe | 46.36% (153) | 48.64% (107) | 43.18% (19) | 40.91% (27) | 45.59% (119) | 44.95% (49) | 35.56% (16) |
| Asia | 53.94% (178) | 57.73% (127) | 54.55% (24) | 40.91% (27) | 59.77% (156) | 58.72% (64) | 55.56% (25) |

Data show the percentage of disease systems found on each continent. In addition to the pathogen being endemic on a continent, the reservoir and target were also required to occur. Many systems (n = 99) were classified as worldwide and are thus classified as being present on each of the listed continents. The number of systems is given in parentheses.

*The epidemic potential zone subsets represent the transmission potential of the pathogen in the target host population(s) following spillover. The zones are defined as follows: dead-end for a basic reproductive number ($R_0$) nearly equal to zero, stuttering chains for an $R_0$ greater than zero but less than one, and epidemic potential for an $R_0$ greater than one.

†High priority zoonotic pathogen subsets were determined by estimating the pathogen's representation in the scientific literature using the H-index. Each subset was created to include pathogens that are among the 25% (Top 25%) and 10% (Top 10%) most significant human pathogens known.
