# Supplementary material for: Are disease reservoirs special? Taxonomic and life history characteristics
Source: PLoS One. 2017 Jul 13;12(7):e0180716. doi: 10.1371/journal.pone.0180716 (PMC5509157; doi:10.1371/journal.pone.0180716)
Supplement: S2 Table — Data show the percentage of systems with each type of transmission between the reservoir and the target. A single system may be categorized as showing more than one transmission type. Trophic transmission included both food- and water-borne transmission. Vector-borne is limited to transmission by arthropods. The number of systems is given in parentheses. *The epidemic potential zone subsets represent the transmission potential of the pathogen in the target host population(s) following spillover. The zones are defined as follows: dead-end for a basic reproductive number (R0) nearly equal to zero, stuttering chains for an R0 greater than zero but less than one, and epidemic potential for an R0 greater than one. †High priority zoonotic pathogen subsets were determined by estimating the pathogen’s representation in the scientific literature using the H-index. Each subset was created to include pathogens that are among the 25% (Top 25%) and 10% (Top 10%) most significant known human pathogens. (PDF) [file pone.0180716.s007.pdf]

S2 Table. Summary of transmission types found in all disease systems and subsets.

		Epidemic Potential Zone*				High Priority Zoonotic Pathogens†	
Transmission Type	All Systems (n = 330)	Dead-End (220)	Stuttering Chains (44)	Epidemic Potential (66)	Human Target (261)	Top 25% (109)	Top 10% (45)
Direct Contact	41.52% (137)	33.18% (73)	54.55% (24)	60.61% (40)	36.78% (96)	37.61% (41)	53.33% (24)
Indirect Contact	15.45% (51)	15.00% (33)	11.36% (5)	19.70% (13)	13.03% (34)	14.68% (16)	8.89% (4)
Vector-Borne	37.88% (125)	36.82% (81)	40.91% (18)	39.39% (26)	39.46% (103)	44.04% (48)	44.44% (20)
Trophic	27.88% (92)	32.73% (71)	27.27% (12)	12.12% (8)	30.27% (79)	27.52% (30)	24.44% (11)

Data show the percentage of systems with each type of transmission between the reservoir and the target. A single system may be categorized as showing more than one transmission type. Trophic transmission included both food- and water-borne transmission. Vector-borne is limited to transmission by arthropods. The number of systems is given in parentheses.

*The epidemic potential zone subsets represent the transmission potential of the pathogen in the target host population(s) following spillover. The zones are defined as follows: dead-end for a basic reproductive number (R_0) nearly equal to zero, stuttering chains for an R_0 greater than zero but less than one, and epidemic potential for an R_0 greater than one.

†High priority zoonotic pathogen subsets were determined by estimating the pathogen's representation in the scientific literature using the H-index. Each subset was created to include pathogens that are among the 25% (Top 25%) and 10% (Top 10%) most significant human pathogens.
