# Supplementary material for: Are disease reservoirs special? Taxonomic and life history characteristics
Source: PLoS One. 2017 Jul 13;12(7):e0180716. doi: 10.1371/journal.pone.0180716 (PMC5509157; doi:10.1371/journal.pone.0180716)
Supplement: S4 Table — The expected distribution represents the number of species in each order that would be identified as reservoirs if they were found as a reservoir host of a human-target pathogen in the same proportions as they are all mammals. Observed numbers total to the 155 mammalian species identified as reservoir hosts. The residual is the absolute difference between expected species and observed species. Standardized residuals are calculated by dividing the residual by the square root of the expected number of species. Positive residuals represent more reservoir species than expected by chance. Negative residuals represent fewer reservoir species than expected by chance. *As listed by Mammal Species of the World [22]. (PDF) [file pone.0180716.s009.pdf]

S4 Table. Observed representation of mammalian orders among reservoirs versus expected for human target systems.

Order	Total Species*	Expected	Observed	Residual	Standardized Residual
Afrosoricida	51	1.46	0	-1.46	-1.21
Artiodactyla	240	6.87	8	1.13	0.43
Carnivora	286	8.19	23	14.81	5.18
Cetacea	84	2.40	0	-2.40	-1.55
Chiroptera	1116	31.94	19	-12.94	-2.29
Cingulata	21	0.60	1	0.40	0.51
Dasyuromorphia	71	2.03	0	-2.03	-1.43
Dermoptera	2	0.06	0	-0.06	-0.24
Didelphimorphia	87	2.49	3	0.51	0.32
Diprotodontia	143	4.09	3	-1.09	-0.54
Erinaceomorpha	24	0.69	3	2.31	2.79
Hyracoidea	4	0.11	2	1.89	5.57
Lagomorpha	92	2.63	6	3.37	2.08
Macroscelidea	15	0.43	0	-0.43	-0.66
Microbiotheria	1	0.03	0	-0.03	-0.17
Monotremata	5	0.14	0	-0.14	-0.38
Notoryctemorphia	2	0.06	0	-0.06	-0.24
Paucituberculata	6	0.17	0	-0.17	-0.41
Peramelemorphia	21	0.60	0	-0.60	-0.78
Perissodactyla	17	0.49	1	0.51	0.74
Pholidota	8	0.23	0	-0.23	-0.48
Pilosa	10	0.29	2	1.71	3.20
Primates	376	10.76	8	-2.76	-0.84
Proboscidea	3	0.09	0	-0.09	-0.29
Rodentia	2277	65.17	72	6.83	0.85
Scandentia	20	0.57	0	-0.57	-0.76
Sirenia	5	0.14	0	-0.14	-0.38
Soricomorpha	428	12.25	4	-8.25	-2.36
Tubulidentata	1	0.03	0	-0.03	-0.17

The expected distribution represents the number of species in each order that would be identified as reservoirs if they were found as a reservoir host of a human-target pathogen in the same proportions as they are all mammals. Observed numbers total to the 155 mammalian species identified as reservoir hosts. The residual is the absolute difference between expected species and observed species. Standardized residuals are calculated by dividing the residual by the square root of the expected number of species. Positive residuals represent more reservoir species than expected by chance. Negative residuals represent fewer reservoir species than expected by chance.

*As listed by *Mammal Species of the World* [22].
