# Supplementary material for: Are disease reservoirs special? Taxonomic and life history characteristics
Source: PLoS One. 2017 Jul 13;12(7):e0180716. doi: 10.1371/journal.pone.0180716 (PMC5509157; doi:10.1371/journal.pone.0180716)
Supplement: S5 Table — (PDF) [file pone.0180716.s010.pdf]

S5 Table. Most represented mammal species among reservoirs.

Order	Species	All Systems (n = 330) Count	Human Target Only (n = 261) Count
Carnivora	*Canis lupus*	44	39
Carnivora	*Felis catus*	37	31
Artiodactyla	*Bos taurus*	32	27
Artiodactyla	*Sus scrofa*	24	21
Artiodactyla	*Ovis aries*	22	16
Rodentia	*Rattus rattus*	18	17
Artiodactyla	*Capra hircus*	16	12
Rodentia	*Rattus norvegicus*	13	12
Carnivora	*Vulpes vulpes*	9	5
Perissodactyla	*Equus caballus*	7	7
Rodentia	*Mus musculus*	7	7
Artiodactyla	*Odocoileus virginianus*	7	4
Rodentia	*Myodes glareolus*	6	6
Rodentia	*Peromyscus leucopus*	6	6
Carnivora	*Canis latrans*	5	3
Artiodactyla	*Syncerus caffer*	5	0
Rodentia	*Cuniculus paca*	4	4
Carnivora	*Procyon lotor*	4	4
Rodentia	*Rhombomys opimus*	4	4
	Others	*157 ≤ 3*	*134 ≤ 3*
