# Supplementary material for: Are disease reservoirs special? Taxonomic and life history characteristics
Source: PLoS One. 2017 Jul 13;12(7):e0180716. doi: 10.1371/journal.pone.0180716 (PMC5509157; doi:10.1371/journal.pone.0180716)
Supplement: S6 Table — Each reservoir was categorized by the orders of mammals represented by the species it included. Orders reflect the hierarchy detailed in Mammal Species of the World [22]. The data above show the percentage of systems whose reservoirs include at least one species belonging to the order. The number of systems is given in parentheses. Unlisted mammalian orders were not found among reservoirs. The ‘All Systems’ and ‘Human Target’ columns are redundant with Table 5 and included here for comparison. (PDF) [file pone.0180716.s011.pdf]

S6 Table. Summary of mammalian reservoirs by orders represented in subsets of viral pathogen systems and viral pathogen systems with human targets.

Order	All Systems (330)	Human Target (261)	Viral Pathogens (112)	Viral + Human Target (82)
Artiodactyla	25.45% (84)	21.07% (55)	18.75% (21)	9.76% (8)
Carnivora	25.15% (83)	25.29% (66)	13.39% (15)	7.32% (6)
Cetacea	0.30% (1)	0.38% (1)	0.00% (0)	0.00% (0)
Chiroptera	2.42% (8)	3.07% (8)	7.14% (8)	9.76% (8)
Cingulata	0.91% (3)	1.15% (3)	0.00% (0)	0.00% (0)
Didelphimorphia	0.61% (2)	0.77% (2)	0.00% (0)	0.00% (0)
Diprotodontia	0.61% (2)	0.38% (1)	0.89% (1)	1.22% (1)
Erinaceomorpha	0.30% (1)	0.38% (1)	0.00% (0)	0.00% (0)
Hyracoidea	0.30% (1)	0.38% (1)	0.00% (0)	0.00% (0)
Lagomorpha	3.03% (10)	2.30% (6)	6.25% (7)	4.88% (4)
Perissodactyla	2.73% (9)	3.07% (8)	0.89% (1)	0.00% (0)
Pilosa	0.61% (2)	0.77% (2)	0.00% (0)	0.00% (0)
Primates	3.03% (10)	3.83% (10)	5.36% (6)	7.32% (6)
Rodentia	29.09% (96)	36.02% (94)	28.57% (32)	37.80% (31)
Soricomorpha	1.21% (4)	1.53% (4)	0.00% (0)	0.00% (0)

Each reservoir was categorized by the orders of mammals represented by the species it included. Orders reflect the hierarchy detailed in *Mammal Species of the World* [22]. The data above show the percentage of systems whose reservoirs include at least one species belonging to the order. The number of systems is given in parentheses. Unlisted mammalian orders were not found among reservoirs. The ‘All Systems’ and ‘Human Target’ columns are redundant with Table 5 and included here for comparison.
