# Supplementary material for: Are disease reservoirs special? Taxonomic and life history characteristics
Source: PLoS One. 2017 Jul 13;12(7):e0180716. doi: 10.1371/journal.pone.0180716 (PMC5509157; doi:10.1371/journal.pone.0180716)
Supplement: S7 Table — The expected distribution represents the number of species in each order that would be identified as reservoirs if they were found as a reservoir host in the same proportions as they are all mammals. Observed numbers are based on the number of unique mammalian species identified as reservoir hosts in each subset (92 in A; 80 in B). Only orders with observed reservoir species in the viral pathogen subset are shown. The residual is the absolute difference between expected species and observed species. Standardized residuals are calculated by dividing the residual by the square root of the expected number of species. Positive residuals represent more reservoir species than expected by chance. Negative residuals represent fewer reservoir species than expected by chance. *As listed by Mammal Species of the World [22]. (PDF) [file pone.0180716.s012.pdf]

S7 Table. Observed representation of unique reservoir species in mammalian orders versus expected for subsets of viral pathogen systems (A) and viral pathogen systems that include humans as a target (B).

A. Viral Pathogen Subset

Order	Total Species*	Expected	Observed	Residual	Standardized Residual
Artiodactyla	240	4.08	12	7.92	3.92
Carnivora	286	4.86	9	4.14	1.88
Chiroptera	1116	18.96	19	0.04	0.01
Diprotodontia	143	2.43	3	0.57	0.37
Lagomorpha	92	1.56	7	5.44	4.35
Perissodactyla	17	0.29	2	1.71	3.18
Primates	376	6.39	6	-0.39	-0.15
Rodentia	2277	38.68	34	-4.68	-0.75

B. Viral Pathogen and Human Target Subset

Order	Total Species*	Expected	Observed	Residual	Standardized Residual
Artiodactyla	240	3.55	6	2.45	1.30
Carnivora	286	4.22	8	3.78	1.84
Chiroptera	1116	16.48	19	2.52	0.62
Diprotodontia	143	2.11	3	0.89	0.61
Lagomorpha	92	1.36	5	3.64	3.12
Perissodactyla	17	0.25	0	-0.25	-0.50
Primates	376	5.55	6	0.45	0.19
Rodentia	2277	33.63	33	-0.63	-0.11

The expected distribution represents the number of species in each order that would be identified as reservoirs if they were found as a reservoir host in the same proportions as they are all mammals. Observed numbers are based on the number of unique mammalian species identified as reservoir hosts in each subset (92 in A; 80 in B). Only orders with observed reservoir species in the viral pathogen subset are shown. The residual is the absolute difference between expected species and observed species. Standardized residuals are calculated by dividing the residual by the square root of the expected number of species. Positive residuals represent more reservoir species than expected by chance. Negative residuals represent fewer reservoir species than expected by chance.

*As listed by *Mammal Species of the World* [22].
